# Melphalan and Curcumin Induce Apoptosis in Retinoblastoma Cells Associated with STAT3 Signaling Modulation

**DOI:** 10.3390/pharmaceutics18050540

**Published:** 2026-04-28

**Authors:** Erkan Duman, Aydın Maçin, İlhan Özdemir, Mehmet Cudi Tuncer

**Affiliations:** 1 Department of Ophthalmology, WestEye Private Hospital, Erbil 44001, Iraq; 2 Department of Ophthalmology, Diyarbakır Private Batı Hospital, 21100 Diyarbakır, Turkey; draydinmacin@outlook.com; 3 Department of Histology and Embryology, Faculty of Medicine, Kahramanmaraş Sütçü İmam University, 46040 Kahramanmaraş, Turkey; ilhanozdemir25@yandex.com; 4 Department of Anatomy, Faculty of Medicine, Dicle University, 21280 Diyarbakır, Turkey; drcudi@hotmail.com

**Keywords:** retinoblastoma, melphalan, curcumin, WERI-Rb-1, STAT3, apoptosis, caspase

## Abstract

**Background/Objectives**: Retinoblastoma treatment remains limited by therapeutic resistance and toxicity. While melphalan is a key chemotherapeutic agent, its efficacy is constrained by adverse effects. Curcumin has emerged as a potential adjunct owing to its capacity to regulate oxidative stress and oncogenic signaling pathways, including STAT3. This study aimed to assess the synergistic tumor-inhibitory effects of melphalan–curcumin combined treatment and to investigate the roles of ROS, apoptosis, and STAT3-associated signaling, including validation in a three-dimensional (3D) tumor spheroid model. **Materials and Methods**: Human retinoblastoma (WERI-Rb-1) and normal keratinocyte (HaCaT) cells were exposed to melphalan, curcumin and the combined treatment regimen. Cell viability was analyzed by MTT assay, and drug interactions were analyzed using the Chou–Talalay method. Migration was evaluated by scratch assay. Intracellular ROS levels were quantified using the DCFH-DA assay and confirmed by flow cytometry. Apoptosis was quantified by Annexin V/PI staining, and caspase activity was assessed colorimetrically and by immunocytochemistry. Cytokine levels were determined by ELISA, and gene expression profiling of STAT3 and apoptosis-associated genes were performed using qRT-PCR. Three-dimensional tumor spheroids were established to evaluate treatment responses in a physiologically relevant model. The contribution of ROS was further investigated using N-acetyl-L-cysteine (NAC) pretreatment. **Results**: The combination of melphalan and curcumin notably reduced WERI-Rb-1 cell viability in a synergistic manner (CI < 1) while exhibiting lower cytotoxicity in HaCaT cells, indicating selective antitumor activity. Co-treatment markedly inhibited cell migration and increased intracellular ROS levels. Cells pretreated with NAC significantly reduced ROS levels accumulation and moderately restored cellular viability, supporting a contributory role of oxidative stress. The combination treatment induced pronounced apoptosis, with increased early and late apoptotic cell populations, enhanced caspase-7 and caspase-9 activity, and elevated caspase-9 protein expression. These effects were associated with upregulation of pro-apoptotic genes (BAX, CASP3, CASP7, CASP9), downregulation of anti-apoptotic genes (BCL2, SURVIVIN), and reduction in STAT3 mRNA expression. In addition, the combination reduced pro-inflammatory cytokine levels. Importantly, these effects were recapitulated in 3D tumor spheroids, where the combination treatment reduced spheroid size and viability and induced structural disruption. NAC-mediated rescue experiments in 3D models further supported the notion that ROS contributes to, but is not solely responsible for, the observed effects. **Conclusions**: Overall, these results suggest that melphalan and curcumin exert synergistic and selective antitumor effects in retinoblastoma cells, associated with changes consistent with ROS-related effects, mitochondrial apoptotic processes, and STAT3-related transcriptional alterations rather than definitive pathway activation. The validation of these effects in a 3D tumor spheroid model provides additional support for the potential clinical significance of this combined treatment; however, additional protein-level and functional validation is required.

## 1. Introduction

Retinoblastoma represents the leading primary intraocular neoplasm affecting children, arising from immature retinal precursor cells [1,2]. Although intra-arterial and intravitreal delivery of chemotherapeutic agents has improved outcomes, therapeutic resistance persists as a major obstacle particularly when vitreous seeding is present and ocular preservation rates in late-stage disease remain unsatisfactory [3,4]. Recent clinical evidence indicates that regimens combining three agents (melphalan, topotecan, and carboplatin) achieve superior globe-salvage outcomes relative to two drug protocols, especially in high-risk cases [5,6]. Among available alkylating agents, melphalan is the cornerstone of intra-arterial therapy and is especially effective against vitreous seeds [7,8]. Nevertheless, monotherapy with melphalan carries notable toxicities including neutropenia, bronchospasm, and vascular injury, and dose escalation is constrained by these limiting adverse effects [9,10]. In parallel, curcumin, a bioactive polyphenol extracted from Curcuma longa rhizomes has demonstrated broad antitumor activity encompassing inhibition of proliferation, promotion of apoptosis, and suppression of metastatic potential across multiple cancer models [11,12]. In experimental retinoblastoma settings, curcumin has been shown to arrest cell division, trigger programmed death, and curtail migratory and invasive behavior, predominantly via interference with the STAT3 signaling axis [13,14,15,16]. This polyphenol also exerts antitumor effects in glioblastoma and head-and-neck squamous carcinoma by blocking STAT3 and stimulating mitochondrial apoptosis through reactive oxygen species (ROS) accumulation [2,17]. In WERI-Rb-1 retinoblastoma cells, concurrent use of curcumin with adriamycin has been documented to amplify apoptotic signaling evidenced by elevated caspase-3 and TP53 transcripts alongside diminished survivin expression [15,18].

Published data on the pharmacological interplay between melphalan and curcumin remain conflicting. At sub-cytotoxic concentrations, curcumin may attenuate the toxicity of alkylating agents through its antioxidant properties. In contrast, at higher concentrations, curcumin can exert pro-oxidant effects, thereby enhancing cytotoxicity and potentially promoting synergistic anticancer activity when combined with alkylating agents [4]. It has been reported that the combined application of curcumin and melphalan in breast cancer cells reduces cell viability more than its use alone, increases ROS levels, and induces apoptosis by activating the caspase cascade [11].

However, the combined effects of melphalan and curcumin in retinoblastoma remain largely unexplored, particularly in the context of mechanistic integration and three-dimensional tumor biology. Based on the complementary pharmacological properties of these agents, we hypothesized that their co-administration would exert enhanced antitumor activity through a multifactorial mechanism involving ROS-associated cellular stress, modulation of STAT3 signaling, and activation of mitochondrial apoptotic pathways, rather than a single dominant pathway.

To address this hypothesis, the present study was conducted to systematically investigate the effects of melphalan and curcumin combination therapy in WERI-Rb-1 retinoblastoma cells and non-malignant HaCaT keratinocytes. We further aimed to investigate the functional contribution of oxidative stress using NAC-based rescue experiments and to validate the identified effects in a three-dimensional tumor spheroid model that better recapitulates tumor architecture and microenvironmental complexity.

## 2. Materials and Methods

### 2.1. Cell Culture Conditions and Maintenance of Retinoblastoma and Keratinocyte Cell Lines

The human retinoblastoma cell line WERI-Rb-1 (ATCC^®^ HTB-169™, Manassas, VA, USA) and the normal human keratinocyte cell line HaCaT (CLS Cell Lines Service, Eppelheim, Germany; catalog no. 300493) were cultured under RPMI-1640 medium (Sigma-Aldrich, St. Louis, MO, USA) supplemented with 10% heat-inactivated fetal bovine serum (FBS; Gibco, Thermo Fisher Scientific, Waltham, MA, USA), 1%.

L-glutamine (Sigma-Aldrich), and 1% penicillin–streptomycin (Gibco, Thermo Fisher Scientific). Cells were maintained at 37 °C in a humidified incubator with 5% CO_2_ (NuAire, Plymouth, MN, USA). HaCaT cells were subcultured using trypsin–EDTA upon reaching approximately 80% confluency, whereas WERI-Rb-1 cells, which grow in suspension, were passaged by dilution under identical culture conditions.

### 2.2. Reagents, Stock Solution Preparation, and Antibody Specifications

Pharmaceutical-grade melphalan (CAS: 148-82-3; purity ≥ 98%) and curcumin (CAS: 458-37-7; purity ≥ 98%) were obtained from Sigma-Aldrich (St. Louis, MO, USA). Stock solutions were prepared by dissolving melphalan in sterile distilled water acidified with 0.1 N HCl, while curcumin was dissolved in sterile distilled water containing 0.1% dimethyl sulfoxide (DMSO). All stock solutions were aliquoted and stored at −20 °C until further use. Primary antibody against caspase-9 and the corresponding horseradish peroxidase (HRP)-conjugated secondary antibody were primary antibodies that were sourced from Santa Cruz Biotechnology (Dallas, TX, USA).

### 2.3. Assessment of Cell Viability and Determination of Cytotoxic Effects Using the MTT Assay

Cell viability was evaluated using the MTT assay based on mitochondrial metabolic activity. WERI-Rb-1 and HaCaT cells were seeded into 96-well plates (Corning Inc., Corning, NY, USA) at appropriate densities and incubated overnight under standard culture conditions.

Cells were then exposed to increasing concentrations of melphalan (1, 2.5, 5, 10, 25, and 50 µM), curcumin (5, 10, 25, 50, 100, and 200 µM), or their combination for 24 and 48 h. Following the treatment period, MTT solution (Sigma-Aldrich, St. Louis, MO, USA) was added to each well and incubated to permit formazan crystal formation.

Following incubation, the supernatant was carefully removed, and the formazan crystals were dissolved using DMSO. Absorbance was measured at 570 nm using a microplate reader (BioTek Instruments, Winooski, VT, USA).

Cell viability was expressed as a percentage relative to untreated control cells. Half maximal inhibitory concentration (IC_50_) values were determined using non-linear regression analysis of dose–response curves in GraphPad Prism software (version 9.0; GraphPad Software, La Jolla, CA, USA). Each condition was tested in triplicate and replicated in at least three independent experiments.

### 2.4. Evaluation of Drug Interaction Using Chou–Talalay Combination Index Analysis

The pharmacological interaction between melphalan and curcumin was analyzed using the Chou–Talalay method, which relies on the median-effect principle. Combination index (CI) values were calculated using CompuSyn software (CompuSyn v1.0, ComboSyn Inc., Paramus, NJ, USA).

For combination analysis, cells were treated with melphalan and curcumin at fixed concentration ratios based on their respective IC_50_ values obtained from dose–response experiments. Cell viability data derived from the MTT assay at 48 h were used as input for the analysis.

The fraction affected (Fa), representing the extent of growth inhibition, was calculated for each treatment condition and used to generate Fa–CI plots. CI values were interpreted as follows: CI < 1 indicates synergism, CI = 1 denotes an additive interaction, whereas CI > 1 indicates antagonism. All analyses were performed using data derived from at least three independent experiments

### 2.5. Determination of Selectivity Index for Cancer-Specific Cytotoxicity

The selectivity of melphalan and curcumin toward cancer cells was determined through calculation of the selectivity index (SI), calculated as the ratio of the IC_50_ value obtained in non-malignant HaCaT cells to that in WERI-Rb-1 retinoblastoma cells.

IC_50_ values used for SI calculation were derived from MTT-based dose–response analyses performed at 48 h. An SI value greater than 3 was considered indicative of preferential cytotoxicity toward tumor cells.

### 2.6. Evaluation of Cell Migration Using Scratch-Wound Assay Under Induced Adherent Conditions

To evaluate the effects of melphalan and curcumin on cell migration, a scratch-wound assay was performed. Although WERI-Rb-1 cells are primarily a suspension cell line, cells were allowed to adhere under experimental conditions to enable transient monolayer formation prior to scratch induction. Only adherent cell populations were used for wound generation and subsequent analysis.

A consistent linear scratch was introduced across the cell monolayer using a sterile 200 µL pipette tip. Detached cells and debris were subsequently removed by gentle washing with phosphate-buffered saline (PBS). Cells were then incubated for 24 h in culture medium containing melphalan (15 µM), curcumin (30 µM), or their combination.

Images of the wound region were acquired at 0 and 24 h using an inverted microscope (Olympus, Tokyo, Japan). Wound closure was quantified with ImageJ software (version 1.53; National Institutes of Health, Bethesda, MD, USA) and expressed as the percentage of gap closure relative to the initial wound width.

### 2.7. Three-Dimensional Tumor Spheroid Modeling to Recapitulate In Vivo-like Drug Responses

To better recapitulate tumor architecture and microenvironmental complexity, three-dimensional (3D) tumor spheroids were generated using the WERI-Rb-1 retinoblastoma cell line.

#### 2.7.1. Establishment of Uniform 3D Tumor Spheroids

A 3D tumor spheroid model was established to evaluate treatment responses under conditions in which mimic cell–cell interactions, nutrient gradients, and diffusion limitations characteristic of solid tumors. WERI-Rb-1 cells were cultured in RPMI-1640 medium supplemented with 10% fetal bovine serum (FBS; Gibco, Thermo Fisher Scientific, Waltham, MA, USA) and 1% penicillin–streptomycin.

Cells in the exponential growth phase were seeded into ultra-low attachment round-bottom 96-well plates (Corning Inc., Corning, NY, USA) at a density of 3 × 10^3^ cells per well in a total volume of 200 µL. The plates were maintained at 37 °C in a humidified incubator with 5% CO_2_ to promote spontaneous cell aggregation and spheroid formation. Uniform and compact spheroids developed within 48–72 h and were subsequently selected for further analyses.

#### 2.7.2. Treatment of 3D Tumor Spheroids Under Diffusion-Limited Conditions

After spheroid formation, the culture medium was carefully exchanged with fresh medium containing melphalan (Sigma-Aldrich, St. Louis, MO, USA), curcumin (Sigma-Aldrich, St. Louis, MO, USA), or their combination. Drug concentrations were selected based on IC_50_ values derived from two-dimensional monolayer dose–response experiments and applied as operational reference concentrations.

To account for reduced proliferation rates and limited drug penetration in 3D structures, spheroids were exposed to treatments for 72 h under standard culture conditions. Vehicle-treated spheroids were included as controls.

#### 2.7.3. Bright-Field Imaging and Qualitative Morphological Assessment

Morphological alterations in spheroids were recorded using an inverted bright-field microscope (Olympus, Tokyo, Japan). Images were captured under identical optical settings for all experimental groups to ensure comparability.

Spheroid morphology was qualitatively assessed based on structural integrity, compactness, and the presence of cellular dispersion or fragmentation.

#### 2.7.4. Quantitative Analysis of Spheroid Size

Spheroid size was measured using ImageJ software (version 1.53; National Institutes of Health, Bethesda, MD, USA). The diameter of each spheroid was determined as the average of two perpendicular measurements taken at the widest points. Analyses were conducted using spheroids obtained from at least three independent biological replicates.

#### 2.7.5. Assessment of 3D Spheroid Viability Using ATP-Based Luminescence Assay

Cell viability in spheroids was assessed using a luminescence-based ATP assay specifically optimized for three-dimensional cultures (CellTiter-Glo^®^ 3D Cell Viability Assay; Promega, Madison, WI, USA).

An equal volume of reagent was added directly to each well to ensure complete spheroid lysis and ATP release. After incubation to allow signal stabilization, luminescence was measured using a microplate reader (BioTek Instruments, Winooski, VT, USA). Viability values were normalized to vehicle-treated controls and expressed as percentages.

#### 2.7.6. Live/Dead Fluorescence Imaging for Spatial Assessment of Cytotoxicity

Treatment-induced cytotoxicity in spheroids was further assessed using fluorescence-based viability staining with Calcein-AM and Ethidium homodimer-1 (Thermo Fisher Scientific, Waltham, MA, USA).

Following staining, spheroids were imaged using an inverted fluorescence microscope (Olympus, Tokyo, Japan) equipped with appropriate excitation and emission filters. Images were acquired at a consistent focal plane and under identical exposure settings across all experimental groups, without post-acquisition processing.

Live/dead staining patterns were interpreted in conjunction with spheroid morphology to assess the spatial distribution of viable and non-viable cells within the three-dimensional tumor structures.

### 2.8. Functional Dissection of ROS Contribution Using NAC-Based Antioxidant Rescue in 3D Tumor Spheroids

The role of ROS in treatment-related cytotoxicity was functionally assessed using an antioxidant rescue strategy involving NAC. Three-dimensional tumor spheroids were generated as described above and maintained until compact, morphologically uniform structures were obtained.

Mature spheroids were pre-exposed to NAC (5 mM; Sigma-Aldrich, St. Louis, MO, USA) for 1 h at 37 °C in a humidified atmosphere containing 5% CO_2_. This condition was selected to facilitate effective intracellular ROS scavenging while minimizing nonspecific cytotoxicity. Following pretreatment, spheroids were treated with melphalan, curcumin, or their combination at concentrations consistent with those used in the 3D spheroid experiments.

Experimental controls consisted of untreated spheroids and spheroids treated with NAC alone. Following treatment initiation, spheroids were maintained under standard culture conditions for the specified durations prior to downstream analyses.

#### 2.8.1. Quantification of Intracellular ROS Levels Following NAC Pretreatment

Intracellular ROS levels were measured using the fluorescent probe 2′,7′-dichlorodihydrofluorescein diacetate (DCFH-DA; Sigma-Aldrich, St. Louis, MO, USA). Following treatment, spheroids were enzymatically dissociated into single-cell suspensions using Accutase^®^ (Sigma-Aldrich) to allow quantitative fluorescence analysis at the cellular level.

Cells were rinsed with PBS and incubated with DCFH-DA (10 µM) for 30 min at 37 °C under light-protected conditions. Following incubation, excess probe was removed by washing with PBS.

Fluorescence intensity was recorded using a microplate reader (λ_excitation = 485 nm, λ_emission = 535 nm). ROS levels were expressed as fold changes relative to untreated controls, enabling comparative evaluation of intracellular oxidative stress across experimental conditions.

#### 2.8.2. Evaluation of Cytotoxicity and Apoptotic Responses Following ROS Scavenging

The effect of ROS neutralization on treatment-induced cytotoxicity was assessed using an ATP-based luminescence assay optimized for three-dimensional spheroid cultures (CellTiter-Glo^®^ 3D Cell Viability Assay; Promega, Madison, WI, USA).

After treatment, an equal volume of reagent was added to each well to achieve complete spheroid disruption and ATP release. Luminescence signals were recorded using a microplate reader, and viability was normalized to untreated control spheroids.

To further elucidate cell death mechanisms, apoptosis was evaluated after spheroid dissociation using Annexin V-FITC/PI staining followed by flow cytometric analysis. The proportions of viable, early apoptotic, late apoptotic, and necrotic cell populations were quantified.

The degree to which NAC pretreatment reduced cytotoxicity and apoptotic cell death was interpreted as a functional indicator of ROS involvement in mediating treatment effects.

### 2.9. Quantitative Assessment of Intracellular ROS Generation in Monolayer Cultures

Intracellular ROS levels were measured using the fluorescent probe 2′,7′-dichlorodihydrofluorescein diacetate (DCFH-DA; Sigma-Aldrich, St. Louis, MO, USA).

WERI-Rb-1 cells were exposed to melphalan (15 µM), curcumin (30 µM), or their combination for the indicated durations. Following treatment, cells were incubated with DCFH-DA (10 µM) for 30 min at 37 °C under light-protected conditions.

Following incubation, cells were washed with PBS to eliminate excess probe. Fluorescence intensity was then measured using a microplate reader (λ_excitation = 485 nm, λ_emission = 535 nm; BioTek Instruments, Winooski, VT, USA). ROS levels were expressed as fold changes relative to vehicle-treated control cells.

### 2.10. Antioxidant Rescue Assay to Evaluate ROS Contribution to Cytotoxicity

To functionally assess the contribution of ROS to treatment-induced cytotoxicity, an antioxidant rescue experiment was performed using N-acetyl-L-cysteine (NAC; Sigma-Aldrich, St. Louis, MO, USA).

Cells were pre-exposed to NAC (5 mM) for 2 h prior to drug treatment to reduce intracellular ROS levels. Following pretreatment, cells were treated with melphalan (15 µM), curcumin (30 µM), or their combination for 48 h under standard culture conditions.

Cell viability was subsequently assessed using the MTT assay, while apoptotic cell death was evaluated by Annexin V-FITC/PI staining followed by flow cytometric analysis. In addition, the enzymatic activities of caspase-7 and caspase-9 were measured as indicators of mitochondrial apoptotic pathway activation.

The extent of NAC-mediated rescue was calculated using the following formula:

Rescue score (%) = [(Viability_NAC + drug − Viability_drug)/(Viability_control − Viability_drug)] × 100

Attenuation of cytotoxicity and apoptosis following NAC pretreatment was interpreted as evidence supporting a contributory, but not exclusive, role of ROS in mediating treatment-induced cell death.

### 2.11. Quantitative Analysis of Apoptotic Cell Death by Annexin V-FITC/PI Flow Cytometry

Apoptotic cell death was quantified using Annexin V-FITC/propidium iodide (PI) dual staining (Thermo Fisher Scientific, Waltham, MA, USA) followed by flow cytometry.

After treatment, WERI-Rb-1 cells were collected and washed twice with ice-cold PBS. The cells were subsequently resuspended in binding buffer and incubated with Annexin V-FITC and PI for 15 min at room temperature under light-protected conditions, following the manufacturer’s protocol.

Following staining, samples were immediately analyzed using a flow cytometer (BD FACSCanto™ II, BD Biosciences, San Jose, CA, USA). At least 20,000 events per sample were acquired to ensure statistical reliability.

Data were analyzed using FlowJo software (version 10.8.1; BD Biosciences, San Jose, CA, USA). Cell populations were classified into four distinct quadrants based on fluorescence signals:

- Viable cells (Annexin V^−^/PI^−^);
- Early apoptotic cells (Annexin V^+^/PI^−^);
- Late apoptotic cells (Annexin V^+^/PI^+^);
- Necrotic cells (Annexin V^−^/PI^+^).

The proportions of each population were quantified and expressed as percentages of the total cell population. All experiments were performed in at least three independent biological replicates.

### 2.12. Quantitative Determination of Caspase-7 and Caspase-9 Enzymatic Activities

The enzymatic activities of caspase-7 and caspase-9 were determined using commercially available colorimetric assay kits (BioVision Inc., Milpitas, CA, USA) in accordance with the manufacturer’s instructions.

Following treatment, WERI-Rb-1 cells (1 × 10^6^ cells per sample) were collected and washed with ice-cold PBS. Cells were lysed on ice for 10 min using the supplied lysis buffer and then centrifuged at 10,000× *g* for 5 min at 4 °C to obtain clarified supernatants.

Protein concentrations were determined using the bicinchoninic acid (BCA) assay (Thermo Fisher Scientific, Waltham, MA, USA). Equal quantities of protein (100 µg) from each sample were then incubated with reaction buffer supplemented with specific chromogenic substrates for caspase-7 and caspase-9.

Samples were incubated at 37 °C for 2 h, and enzymatic activity was quantified by measuring absorbance at 405 nm using a microplate reader (BioTek Instruments, Winooski, VT, USA).

Caspase activities were normalized to untreated control samples and expressed as fold change.

All experiments were performed in at least three independent biological replicates.

### 2.13. Immunocytochemical Detection and Quantitative Analysis of Caspase-9 Expression

Immunocytochemical analysis of caspase-9 expression was performed to evaluate protein-level changes associated with treatment-induced apoptosis in WERI-Rb-1 cells.

Cells (1 × 10^5^ per well) were plated on poly-L-lysine-coated glass coverslips and permitted to adhere for 24 h. Following 48 h of treatment, cells were fixed in 4% paraformaldehyde (Sigma-Aldrich, St. Louis, MO, USA) for 15 min at room temperature and subsequently permeabilized with 0.1% Triton X-100 (Sigma-Aldrich) for 10 min.

Endogenous peroxidase activity was inhibited by treatment with 3% hydrogen peroxide (H_2_O_2_) for 10 min. Non-specific binding was blocked using 5% bovine serum albumin (BSA; Sigma-Aldrich) for 30 min at room temperature.

Cells were incubated overnight at 4 °C with an anti-caspase-9 primary antibody (Santa Cruz Biotechnology, Dallas, TX, USA; 1:200 dilution), followed by incubation with an HRP-conjugated secondary antibody (Santa Cruz Biotechnology; 1:500 dilution) for 1 h at room temperature.

Immunoreactivity was detected using 3,3′-diaminobenzidine (DAB) substrate (Vector Laboratories, Newark, CA, USA), and nuclei were counterstained with Mayer’s hematoxylin.

Stained cells were examined and imaged using an inverted light microscope (Olympus, Tokyo, Japan) equipped with a digital imaging system under identical acquisition settings for all experimental groups.

Caspase-9 expression was assessed using both semi-quantitative and quantitative methods. Staining intensity was categorized as weak (+), moderate (++), or strong (+++). In addition, quantitative analysis was conducted by calculating integrated optical density (IOD) values using ImageJ software (version 1.53; National Institutes of Health, Bethesda, MD, USA). All measurements were performed on at least three independent biological replicates.

### 2.14. Quantitative Measurement of Pro-Inflammatory Cytokines by ELISA

The levels of the pro-inflammatory cytokines interleukin-6 (IL-6), interleukin-1β (IL-1β), and tumor necrosis factor-α (TNF-α) released into the culture medium were quantified using sandwich enzyme-linked immunosorbent assay (ELISA) kits (eBioscience, San Diego, CA, USA) following the manufacturer’s instructions.

WERI-Rb-1 cells (1 × 10^6^ cells per condition) were treated with melphalan (15 µM), curcumin (30 µM), or their combination for 48 h under standard culture conditions.

Following treatment, culture supernatants were collected and centrifuged at 3000 rpm for 10 min at 4 °C to eliminate cellular debris. The clarified supernatants were then used for cytokine quantification. ELISAs were performed according to the manufacturer’s protocol, and absorbance was measured at 450 nm using a microplate reader (BioTek Instruments, Winooski, VT, USA).

Cytokine levels were calculated based on standard curves and reported as pg/mL. To reduce potential bias associated with treatment-related differences in cell viability, cytokine data were evaluated in conjunction with corresponding viability measurements. All experiments were performed using a minimum of three independent biological replicates.

### 2.15. Quantitative Gene Expression Analysis by Real-Time PCR (qRT-PCR)

RNA isolation was performed on treated and control WERI-Rb-1 cells using a commercial kit (Qiagen RNeasy Mini Kit, Qiagen, Hilden, Germany) according to the manufacturer’s guidelines.

RNA purity and concentration were determined spectrophotometrically using a NanoDrop™ instrument (Thermo Fisher Scientific, Waltham, MA, USA). Complementary DNA (cDNA) was synthesized from 1 µg of total RNA using a reverse transcription kit (High-Capacity cDNA Reverse Transcription Kit, Applied Biosystems, Foster City, CA, USA).

Quantitative real-time PCR (qRT-PCR) was conducted using a SYBR Green Master Mix (Applied Biosystems, Foster City, CA, USA) on a StepOnePlus™ real-time PCR system (Applied Biosystems). Each reaction was performed in a final volume of 20 µL, containing cDNA template, gene-specific primers, and SYBR Green PCR Master Mix.

The expression levels of apoptosis- and signaling-related genes, including BAX, BCL2, CASP3, CASP7, CASP9, STAT3, and survivin, were analyzed.

Gene expression levels were normalized using β-actin (ACTB) and GAPDH as internal reference genes. Relative expression levels were calculated using the 2^−ΔΔCt^ method.

All reactions were performed in triplicate, and no-template controls were included to exclude contamination.

Primer sequences are provided in Table 1.

PCR amplification was performed with an initial denaturation at 95 °C for 10 min, followed by 40 cycles of 95 °C for 15 s and 60 °C for 1 min. Relative gene expression was calculated using the 2^−ΔΔCt^ method and normalized against ACTB and GAPDH as internal controls, with the geometric mean of these reference genes applied for normalization.

### 2.16. Bioinformatic Analyses

#### 2.16.1. Identification of Drug-Associated Target Genes and Data Integration Strategy

A bioinformatics-based workflow was employed to identify putative molecular targets of melphalan and curcumin in the context of retinoblastoma.

Target genes associated with curcumin were collected from the PubChem (https://pubchem.ncbi.nlm.nih.gov/ accessed 10 February 2026) and STRING (version 12.0; https://string-db.org/ accessed 10 February 2026) databases, while genes related to melphalan were sourced from the Comparative Toxicogenomics Database (CTD; http://ctdbase.org/ accessed 10 February 2026).

Only human (*Homo sapiens*) gene entries were included in the analysis. Duplicate entries were removed, and gene lists were curated prior to downstream analysis.

The resulting gene sets were subsequently used for comparative analysis to identify shared targets between melphalan and curcumin.

#### 2.16.2. Protein–Protein Interaction (PPI) Network Construction and Topological Analysis

PPI networks among the shared target genes were constructed using the STRING database (version 12.0; https://string-db.org/ accessed 10 February 2026) with a medium-confidence interaction threshold (confidence score ≥ 0.4).

The resulting interaction network was imported into Cytoscape software (version 3.10.0; Cytoscape Consortium, San Diego, CA, USA) for visualization and further analysis.

Topological parameters, including degree centrality (node connectivity), closeness centrality, and betweenness centrality, were calculated using the NetworkAnalyzer tool in Cytoscape to evaluate the relative importance of individual nodes within the network.

Genes with the highest degree centrality values were identified as hub candidates. The top ten genes with the highest connectivity were selected as putative hub proteins for downstream interpretation.

#### 2.16.3. Gene Ontology (GO) and Kyoto Encyclopedia of Genes and Genomes (KEGG) Pathway Enrichment Analysis

Functional enrichment analysis was performed to explore the biological relevance of the shared target genes identified between melphalan and curcumin.

Gene Ontology (GO) enrichment analysis was performed to identify significantly overrepresented biological processes (BP), cellular components (CC), and molecular functions (MF). Kyoto Encyclopedia of Genes and Genomes (KEGG) pathway analysis was conducted to determine enriched signaling pathways associated with the target gene set.

Enrichment analyses were carried out using the Database for Annotation, Visualization and Integrated Discovery (DAVID, version 6.8; https://davidbioinformatics.nih.gov/ accessed 10 February 2026) with *Homo sapiens* selected as the background species.

Statistical significance was evaluated using a modified Fisher’s exact test (EASE score), and *p*-values were adjusted for multiple testing using the Benjamini–Hochberg procedure. Terms with an adjusted *p*-value below 0.05 and an FDR < 0.05 were deemed statistically significant.

#### 2.16.4. In Silico Exploration of STAT3-Associated Gene Expression Patterns in Retinoblastoma

To explore the expression patterns of STAT3 and apoptosis-related genes, publicly available retinoblastoma transcriptomic datasets (GSE110811 and GSE97508) were retrieved from the Gene Expression Omnibus (GEO) database (https://www.ncbi.nlm.nih.gov/geo/ accessed 10 February 2026).

Gene expression levels of STAT3, BCL2, BAX, CASP3, CASP7, CASP9, and survivin (BIRC5) were extracted and compared between retinoblastoma samples and normal retinal tissues.

Differential expression analysis was performed using normalized expression values provided within the datasets. In addition, Pearson correlation analysis was conducted to assess the relationships between STAT3 and apoptosis-related genes.

These analyses were performed to provide supportive, hypothesis-generating insights into potential associations between STAT3 signaling and apoptotic regulation in retinoblastoma.

### 2.17. Statistical Analysis of Experimental Data

All experiments were independently conducted at least three times (*n* = 3 biological replicates), with each biological replicate comprising a minimum of three technical repeats. Data are presented as mean ± standard deviation (SD). Statistical analyses were performed using GraphPad Prism software (version 9.0; GraphPad Software, La Jolla, CA, USA). Comparisons between two groups were analyzed using an unpaired Student’s *t*-test, while multiple group comparisons were performed using one-way analysis of variance (ANOVA) followed by Tukey’s post hoc test for pairwise comparisons. Assumptions of normality and homogeneity of variance were assessed prior to analysis. Differences were considered statistically significant at *p* < 0.05. Statistical significance was denoted as follows: * *p* < 0.05, ** *p* < 0.01, *** *p* < 0.001.

## 3. Results

### 3.1. Melphalan and Curcumin Reduce Cell Viability with Preferential Cytotoxicity in Retinoblastoma Cells

**Figure 1:**
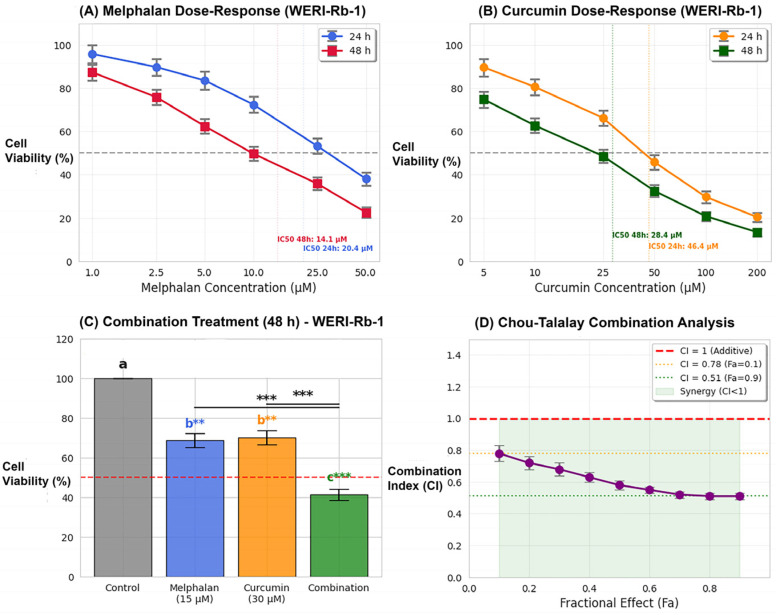
Dose-dependent growth inhibition by melphalan and curcumin in WERI-Rb-1 cells. (**A**) Cell viability curves for melphalan at 24 and 48 h. Cells were treated with increasing concentrations of melphalan (1–50 µM), and viability was assessed using the MTT assay. Cell viability was calculated as the percentage of viable cells relative to the untreated control group, which was defined as 100%, using the formula: (treated absorbance/control absorbance) × 100. (**B**) Dose–response curves for curcumin at 24 and 48 h. Cells were exposed to increasing concentrations of curcumin (5–200 µM), and cell viability was determined by MTT assay. Cell viability was calculated as described above. (**C**) Effects of combination treatment at 48 h. Cells were treated with melphalan (15 µM), curcumin (30 µM), or their combination. The combination treatment significantly reduced cell viability (41.3 ± 2.7%) compared with melphalan alone (68.7 ± 3.4%) and curcumin alone (70.2 ± 3.5%) (** *p* < 0.01, *** *p* < 0.001). Different letters (a–c) indicate statistically significant differences between groups (*p* < 0.05), whereas groups sharing the same letter are not significantly different. (**D**) Chou–Talalay CI analysis for melphalan–curcumin interaction. CI values were calculated from 48 h viability data using CompuSyn software (ComboSyn Inc., USA). CI values ranged from 0.51 to 0.78 across fractional effect (Fa) levels (0.1–0.9), indicating a synergistic interaction (CI < 1), with stronger synergy observed at higher effect levels. Data are presented as mean ± SD (*n* = 3 independent experiments). Statistical analysis for panel (**C**) was performed using one-way ANOVA followed by Tukey’s post hoc test.

Growth-inhibitory activity was evaluated by exposing WERI-Rb-1 cells to increasing concentrations of melphalan (1, 2.5, 5, 10, 25, and 50 µM) and curcumin (5, 10, 25, 50, 100, and 200 µM) for 24 and 48 h. MTT analysis demonstrated that both agents induced concentration- and time-dependent reductions in cell viability (*p* < 0.05–0.001). For melphalan treatment, cell viability at 24 h decreased from 95.8 ± 4.2% (1 µM) to 38.1 ± 3.1% (50 µM). At 48 h, a more pronounced reduction was observed, with viability declining from 87.3 ± 3.7% (1 µM) to 22.6 ± 2.4% (50 µM) (Figure 1A). Similarly, curcumin treatment resulted in a dose-dependent decrease in viability. At 24 h, viability decreased from 89.6 ± 4.0% (5 µM) to 20.4 ± 2.2% (200 µM), whereas at 48 h, values declined further from 74.8 ± 3.7% to 13.4 ± 1.8%, respectively (Figure 1B). Based on IC_50_ values derived from dose–response curves, combination experiments were performed using melphalan (15 µM) and curcumin (30 µM). After 48 h treatment, melphalan alone reduced cell viability to 68.7 ± 3.4%, and curcumin alone to 70.2 ± 3.5%, whereas combination treatment resulted in a marked reduction to 41.3 ± 2.7% (*p* < 0.001 vs. single treatments) (Figure 1C). Pharmacodynamic interaction between melphalan and curcumin was further evaluated using the Chou–Talalay CI method. The CI value at the tested concentration was 0.78, indicating a synergistic interaction (CI < 1). Analysis across fractional effect (Fa) levels revealed CI values ranging from 0.51 to 0.78 (Fa = 0.1–0.9), suggesting increasing synergy at higher effect levels (Figure 1D). IC_50_ values calculated by non-linear regression analysis were 20.4 µM (24 h) and 14.1 µM (48 h) for melphalan, and 46.4 µM (24 h) and 28.4 µM (48 h) for curcumin. For both agents, IC_50_ values at 48 h were significantly lower than those at 24 h (*p* < 0.05 for melphalan; *p* < 0.01 for curcumin), indicating enhanced cytotoxicity with prolonged exposure.

**Figure 2:**
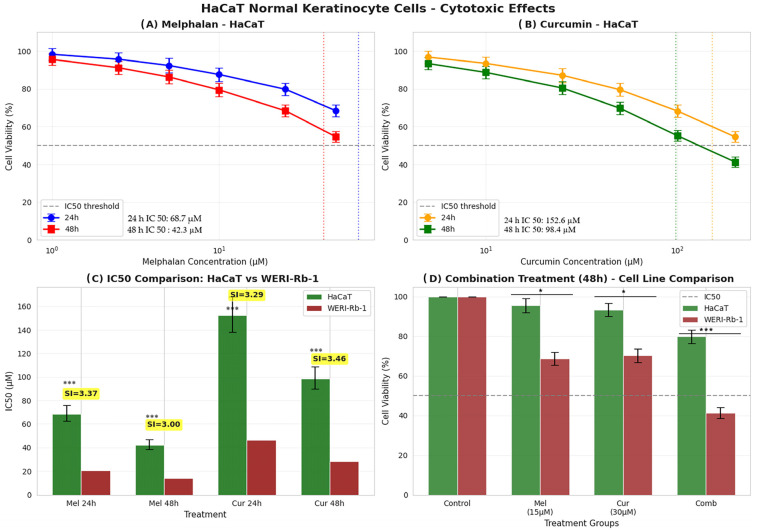
Differential cytotoxic effects of melphalan and curcumin in HaCaT keratinocytes compared with WERI-Rb-1 retinoblastoma cells. (**A**) Dose–response curves of melphalan in HaCaT cells at 24 and 48 h. Cell viability decreased in a concentration- and time-dependent manner, with IC_50_ values of 68.7 µM (24 h) and 42.3 µM (48 h). Cell viability was assessed using the MTT assay and calculated as the percentage of viable cells relative to the untreated control group, defined as 100%, using the formula: (treated absorbance/control absorbance) × 100. (**B**) Dose–response curves of curcumin in HaCaT cells at 24 and 48 h. IC_50_ values were determined as 152.6 µM (24 h) and 98.4 µM (48 h). Cell viability was calculated as described above. (**C**) Comparative IC_50_ analysis between HaCaT and WERI-Rb-1 cells. SI was calculated as the ratio of IC_50_ values in HaCaT cells to those in WERI-Rb-1 cells, with SI > 3 indicating preferential cytotoxicity toward tumor cells (*** *p* < 0.001). (**D**) Cell viability comparison following 48 h treatment with melphalan (15 µM), curcumin (30 µM), and their combination. Combination treatment markedly reduced viability in WERI-Rb-1 cells (41.3 ± 2.7%) while HaCaT cells retained higher viability (79.8 ± 3.5%). Data are presented as mean ± SD (*n* = 3 independent experiments). Statistical analysis for panel (**D**) was performed using one-way ANOVA followed by Tukey’s post hoc test. * *p* < 0.05, *** *p* < 0.001.

To evaluate treatment selectivity, non-malignant HaCaT keratinocytes were exposed to identical experimental conditions, and cell viability was assessed using the MTT assay. Both melphalan and curcumin exhibited substantially lower cytotoxic effects in HaCaT cells compared with WERI-Rb-1 retinoblastoma cells (*p* < 0.001), indicating preferential activity toward malignant cells. Dose–response analysis demonstrated that melphalan reduced HaCaT cell viability in a concentration- and time-dependent manner, with IC_50_ values of 68.7 µM at 24 h and 42.3 µM at 48 h (Figure 2A). Similarly, curcumin treatment resulted in IC_50_ values of 152.6 µM at 24 h and 98.4 µM at 48 h (Figure 2B). These values were consistently higher than those observed in WERI-Rb-1 cells, confirming reduced sensitivity of normal keratinocytes to both agents. Comparative IC_50_ analysis revealed SI values exceeding 3 for both compounds across treatment conditions, supporting a favorable therapeutic window and preferential cytotoxicity toward retinoblastoma cells (Figure 2C). Following 48 h exposure, combination treatment with melphalan (15 µM) and curcumin (30 µM) resulted in a marked reduction in WERI-Rb-1 cell viability (41.3 ± 2.7%), whereas HaCaT cells retained significantly higher viability (79.8 ± 3.5%) (*p* < 0.001; Figure 2D). These findings demonstrate that the combination treatment exerts enhanced cytotoxic effects in tumor cells while relatively sparing non-malignant cells.

### 3.2. Combination Treatment Inhibits Migration of WERI-Rb-1 Cells

**Figure 3:**
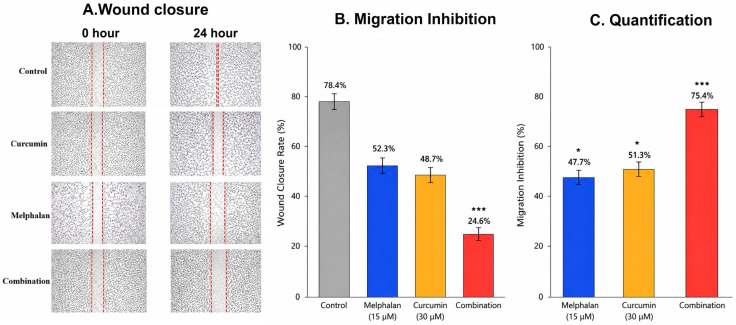
Inhibitory effects of melphalan and curcumin on WERI-Rb-1 cell migration assessed by scratch-wound assay. (**A**) Representative images of wound closure at 0 and 24 h following treatment with melphalan, curcumin, and their combination. Red dashed lines indicate the initial wound boundaries used for the assessment of wound closure. Control cells exhibited substantial wound closure, whereas treated groups showed reduced migratory capacity, most prominently in the combination group. (**B**) Quantitative analysis of wound closure rate (%). Control cells displayed 78.4 ± 5.2% closure, whereas melphalan and curcumin treatments reduced closure to 52.3 ± 4.1% and 48.7 ± 3.8%, respectively. Combination treatment resulted in the lowest wound closure (24.6 ± 3.1%). Wound closure (%) was calculated as: [(initial wound area − remaining wound area at 24 h)/initial wound area] × 100. (**C**) Migration inhibition percentages calculated relative to control. Combination treatment showed the strongest inhibition (75.4%), compared with melphalan (47.7%) and curcumin (51.3%). Migration inhibition (%) was calculated relative to the control group using wound closure values. Data are presented as mean ± SD (*n* = 3 independent experiments). Statistical analysis was performed using one-way ANOVA followed by Tukey’s post hoc test (* *p* < 0.05, *** *p* < 0.001).

The impact of melphalan and curcumin on cellular motility was evaluated using a scratch-wound assay in WERI-Rb-1 cells. Cells were treated with melphalan (15 µM), curcumin (30 µM), or their combination for 24 h. In the control group, 78.4 ± 5.2% of the wound area was closed after 24 h. Treatment with melphalan reduced wound closure to 52.3 ± 4.1% (*p* < 0.01 vs. control), while curcumin further decreased closure to 48.7 ± 3.8% (*p* < 0.001 vs. control) (Figure 3). Combination treatment produced the strongest inhibitory effect, reducing wound closure to 24.6 ± 3.1%, which was significantly lower than both melphalan and curcumin alone (*p* < 0.001 for both comparisons) (Figure 3). Consistent with these findings, migration inhibition analysis demonstrated that the combination treatment achieved the highest inhibition rate (75.4%), compared with melphalan (47.7%) and curcumin (51.3%), indicating a markedly enhanced suppression of cell motility by the combined regimen.

### 3.3. Melphalan–Curcumin Co-Treatment Enhances ROS Generation and Is Partially Reversed by NAC

**Figure 4:**
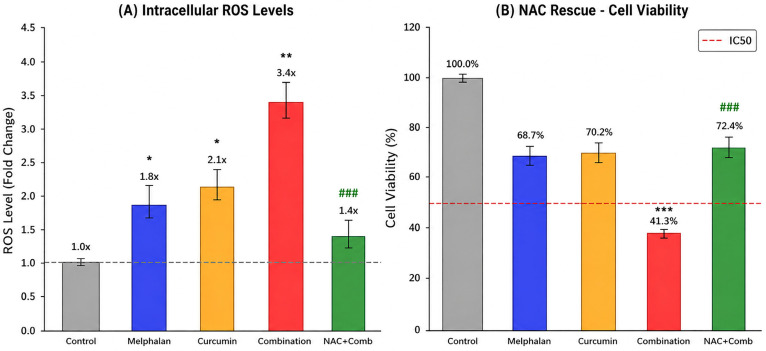
Oxidative stress-associated cytotoxic effects of melphalan–curcumin treatment in WERI-Rb-1 cells. (**A**) Intracellular ROS levels measured by DCFH-DA fluorescence following 24 h treatment. Melphalan and curcumin increased ROS levels, with the combination treatment inducing the highest oxidative stress. NAC pretreatment attenuated ROS accumulation. ROS levels are expressed as fold change relative to the untreated control group. (**B**) NAC rescue experiment showing cell viability after 48 h treatment. Combination treatment markedly reduced viability, whereas NAC pretreatment increased cell viability in the combination group, indicating a partial reversal of cytotoxic effects. Cell viability was calculated as the percentage of viable cells relative to the untreated control group, defined as 100%, using the formula: (treated signal/control signal) × 100. Data are presented as mean ± SD (*n* = 3 independent experiments). Statistical significance: * *p* < 0.05, ** *p* < 0.01, *** *p* < 0.001 vs. control; ### *p* < 0.001 vs. combination (one-way ANOVA followed by Tukey’s post hoc test).

Intracellular oxidative stress was quantified in WERI-Rb-1 cells using DCFH-DA fluorescence analysis. Compared with control cells, melphalan (15 µM) treatment increased ROS levels by 1.8-fold (*p* < 0.05), while curcumin (30 µM) induced a 2.1-fold increase (*p* < 0.01). Combination treatment resulted in a marked elevation of ROS levels to 3.4-fold relative to control, which was significantly higher than both single treatments (*p* < 0.001; Figure 4A). Importantly, pretreatment with the ROS scavenger NAC substantially reduced ROS accumulation in the combination group, decreasing ROS levels to approximately 1.4-fold relative to control (*p* < 0.001 vs. combination). These findings indicate that melphalan and curcumin cooperatively enhance intracellular oxidative stress, and that this effect is at least partially reversible by antioxidant intervention, supporting a contributory role of ROS in the observed cytotoxic response.

**Figure 5:**
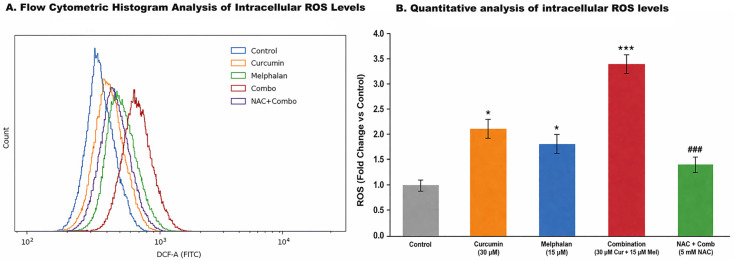
Flow cytometric and quantitative analysis of intracellular ROS levels in WERI-Rb-1 cells. (**A**) Flow cytometric histogram analysis of intracellular ROS levels measured using DCFH-DA staining following 48 h treatment with curcumin (30 µM), melphalan (15 µM), their combination, or NAC pretreatment. Histograms represent fluorescence intensity distributions (DCF-A, FITC channel) corresponding to intracellular ROS levels at the single-cell level. Compared with control cells, both curcumin and melphalan treatments induced a rightward shift in fluorescence intensity, indicating increased intracellular ROS levels. The combination treatment resulted in the most pronounced shift, whereas NAC pretreatment partially reversed this effect. (**B**) Quantitative analysis of intracellular ROS levels expressed as fold change relative to control. Combination treatment significantly increased ROS levels compared with single treatments, while NAC pretreatment significantly attenuated ROS accumulation in the combination group. Data are presented as mean ± SD (*n* = 3). Statistical analysis was performed using one-way ANOVA followed by Tukey’s post hoc test (* *p* < 0.05, *** *p* < 0.001 vs. control; ### *p* < 0.001 vs. combination).

To further characterize intracellular ROS accumulation at the single-cell level, flow cytometric histogram analysis of DCF fluorescence was performed. Compared with control cells, both curcumin and melphalan treatments induced a rightward shift in fluorescence intensity distributions, indicating increased intracellular ROS production. This shift was more pronounced in the combination-treated group, demonstrating a substantial elevation in ROS levels across the cell population. In contrast, NAC pretreatment partially reversed this shift, with fluorescence intensity distributions moving toward control levels, consistent with effective ROS scavenging. These findings corroborate the fluorometric ROS measurements and support a ROS-associated contribution to the cytotoxic effects observed following melphalan and curcumin co-treatment (Figure 5).

To evaluate the functional contribution of ROS to treatment-induced cytotoxicity, NAC-mediated rescue experiments were performed. As shown in Figure 4B, combination treatment markedly reduced cell viability to 41.3 ± 2.7%. NAC pretreatment significantly increased cell viability in the combination group to approximately 72–75% (*p* < 0.001 vs. combination alone), indicating a substantial rescue effect. However, NAC did not fully restore viability to control levels, suggesting that ROS contributes significantly, but not exclusively, to the cytotoxic effects induced by melphalan and curcumin co-treatment.

### 3.4. Combination Treatment Induces Apoptosis and Pro-Apoptotic Gene Expression Changes

**Figure 6:**
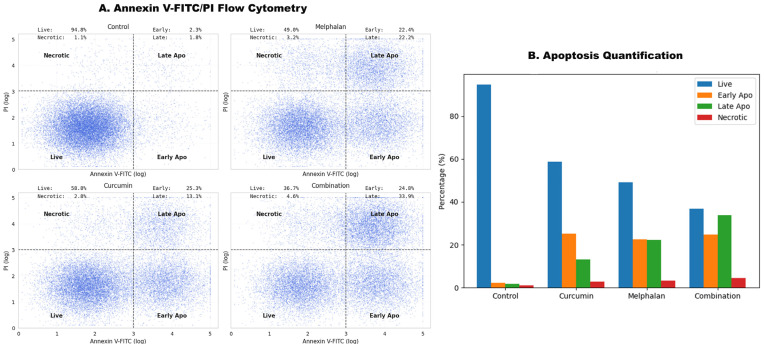
Apoptosis analysis in WERI-Rb-1 retinoblastoma cells by Annexin V-FITC/PI staining and flow cytometry following 48 h treatment. (**A**) Representative flow cytometry dot plots (20,000 events per sample) showing apoptosis distribution in cells treated with melphalan (15 µM), curcumin (30 µM), their combination, or vehicle control. Quadrants indicate: lower-left (live cells: Annexin V^−^/PI^−^), lower-right (early apoptotic cells: Annexin V^+^/PI^−^), upper-right (late apoptotic cells: Annexin V^+^/PI^+^), and upper-left (necrotic cells: Annexin V^−^/PI^+^). Both melphalan and curcumin treatments increased apoptotic cell populations compared with control, while combination treatment induced the highest levels of apoptosis, characterized by a marked increase in both early and late apoptotic fractions. Percentages in each quadrant are representative of three independent experiments. (**B**) Quantitative distribution of cell populations (live, early apoptotic, late apoptotic, and necrotic cells) presented as a bar graph based on flow cytometry analysis. Data are presented as mean ± SD (*n* = 3).

Apoptotic responses to drug treatment were evaluated using Annexin V-FITC/PI staining followed by flow cytometry in WERI-Rb-1 cells after 48 h exposure to melphalan (15 µM), curcumin (30 µM), or their combination. In the control group, viable cells accounted for 94.8 ± 2.1%, while early apoptotic, late apoptotic, and necrotic populations were 2.3 ± 0.4%, 1.8 ± 0.3%, and 1.1 ± 0.2%, respectively. Melphalan treatment increased total apoptosis (early + late) to 44.6 ± 3.2% (*p* < 0.01 vs. control), with early apoptotic cells at 22.4 ± 2.1% and late apoptotic cells at 22.2 ± 2.0%. Necrotic cells accounted for 3.2 ± 0.5%. Curcumin treatment elevated total apoptosis to 38.4 ± 3.0% (*p* < 0.001 vs. control), with early apoptotic cells at 25.3 ± 2.4% and late apoptotic cells at 13.1 ± 1.6%, while necrosis remained limited (2.8 ± 0.4%). Combination treatment induced the highest apoptotic response, with total apoptosis reaching 58.7 ± 4.2% (*p* < 0.001 vs. control, melphalan, and curcumin). Early and late apoptotic fractions were 24.8 ± 2.3% and 33.9 ± 3.1%, respectively. Necrotic cell levels showed a modest increase (4.6 ± 0.7%) but did not differ significantly from single treatments (*p* > 0.05) (Figure 6).

**Figure 7:**
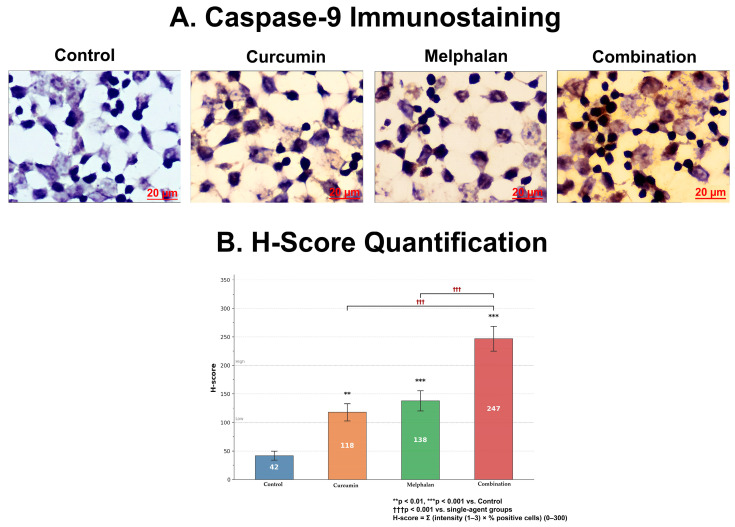
Immunocytochemical evaluation of caspase-9 protein levels in WERI-Rb-1 cells. (**A**) Representative DAB/hematoxylin-stained photomicrographs showing caspase-9 immunoreactivity (40×). Control cells exhibit weak cytoplasmic staining, indicating low basal caspase-9 protein levels. Curcumin- and melphalan-treated cells display increased cytoplasmic staining of moderate to strong intensity. Combination-treated cells show the highest level of cytoplasmic immunoreactivity, consistent with increased caspase-9 protein expression. Scale bar = 20 µm. (**B**) Quantitative analysis of caspase-9 immunoreactivity expressed as H-score. Cells were treated with melphalan (15 µM), curcumin (30 µM), or their combination for 48 h. Data are presented as mean ± SD (*n* = 3). Statistical significance was determined by one-way ANOVA followed by Tukey’s post hoc test. ** *p* < 0.01, *** *p* < 0.001 vs. control; ††† *p* < 0.001 vs. single-agent treatment groups.

Caspase-9 protein levels were evaluated by immunocytochemical staining in WERI-Rb-1 cells following 48 h treatment with melphalan (15 µM), curcumin (30 µM), or their combination. In control cells, weak cytoplasmic immunoreactivity was observed, with IOD value of 42 ± 12. Curcumin treatment increased caspase-9 immunoreactivity to moderate levels (118 ± 18; *p* < 0.01 vs. control), while melphalan treatment produced a more pronounced increase (138 ± 22; *p* < 0.001 vs. control). Notably, combination treatment resulted in the strongest caspase-9 immunoreactivity, characterized by intense cytoplasmic staining and a significantly elevated IOD value (247 ± 35), exceeding both single-agent treatments (*p* < 0.001). These findings indicate that combined melphalan and curcumin treatment is associated with enhanced caspase-9 protein accumulation, consistent with activation of mitochondrial-associated apoptotic signaling pathways (Figure 7).

**Figure 8:**
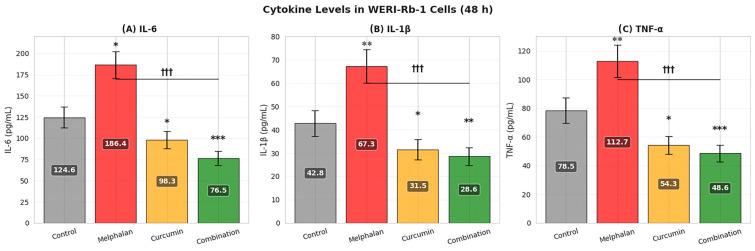
Differential modulation of pro-inflammatory cytokines in WERI-Rb-1 cells following melphalan and curcumin treatment. Levels of IL-6 (**A**), IL-1β (**B**), and TNF-α (**C**) were measured by ELISA after 48 h exposure to melphalan (15 µM), curcumin (30 µM), their combination, or vehicle control. Melphalan treatment increased cytokine levels, whereas curcumin reduced cytokine production relative to control. Combination treatment resulted in the lowest cytokine levels, suggesting attenuation of melphalan-induced inflammatory responses. Data are presented as mean ± SD (*n* = 3). Statistical analysis was performed using one-way ANOVA followed by Tukey’s post hoc test. * *p* < 0.05, ** *p* < 0.01, *** *p* < 0.001 vs. control; ††† *p* < 0.001 vs. melphalan.

Concentrations of IL-6, IL-1β, and TNF-α were quantified in WERI-Rb-1 cell-conditioned media by ELISA following 48 h treatment. In control cells, baseline cytokine levels were 124.6 ± 12.3 pg/mL for IL-6, 42.8 ± 5.6 pg/mL for IL-1β, and 78.5 ± 8.9 pg/mL for TNF-α. Melphalan treatment significantly increased all measured cytokines (IL-6: 186.4 ± 15.7 pg/mL, *p* < 0.05; IL-1β: 67.3 ± 7.2 pg/mL, *p* < 0.01; TNF-α: 112.7 ± 11.4 pg/mL, *p* < 0.01), indicating induction of a pro-inflammatory response. In contrast, curcumin treatment reduced cytokine levels below control values (IL-6: 98.3 ± 10.2 pg/mL; IL-1β: 31.5 ± 4.3 pg/mL; TNF-α: 54.3 ± 6.2 pg/mL; *p* < 0.05), consistent with its anti-inflammatory properties. Importantly, combination treatment resulted in the lowest cytokine levels across all groups (IL-6: 76.5 ± 8.4 pg/mL; IL-1β: 28.6 ± 3.9 pg/mL; TNF-α: 48.6 ± 5.8 pg/mL), significantly lower than melphalan alone (*p* < 0.001). These findings indicate that curcumin effectively counteracts melphalan-induced inflammatory signaling, and that the combined treatment shifts the cytokine profile toward a suppressed inflammatory state (Figure 8).

**Figure 9:**
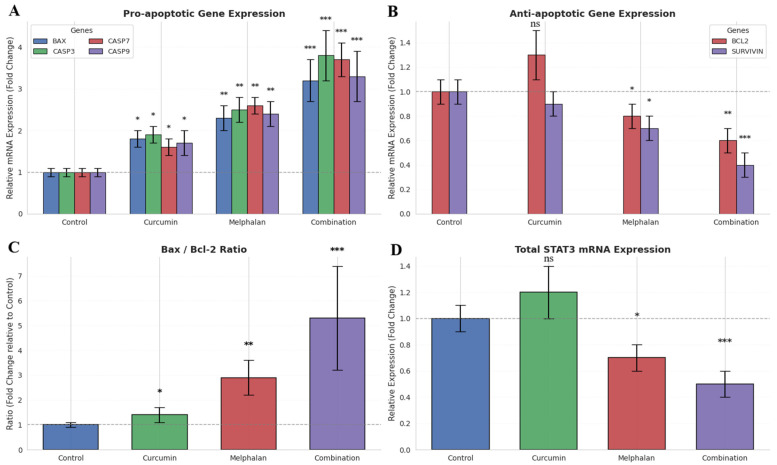
Transcriptional changes in apoptotic and STAT3-related pathways in WERI-Rb-1 cells following melphalan and curcumin treatment. (**A**) Relative mRNA expression of pro-apoptotic genes (BAX, CASP3, CASP7, CASP9). (**B**) Relative mRNA expression of anti-apoptotic genes (BCL2 and SURVIVIN). (**C**) Bax/Bcl-2 ratio indicating the balance between pro- and anti-apoptotic regulation. (**D**) Relative STAT3 mRNA expression levels. Cells were treated with curcumin (30 µM), melphalan (15 µM), their combination, or vehicle control for 48 h. Combination treatment resulted in enhanced pro-apoptotic gene expression, reduced expression of anti-apoptotic genes, an increased Bax/Bcl-2 ratio, and decreased STAT3 mRNA levels, suggesting a shift toward a pro-apoptotic transcriptional profile rather than direct evidence of pathway modulation. Data are presented as mean ± SD (*n* = 3), expressed as fold change relative to control and normalized to β-actin and GAPDH using the 2^−ΔΔCt^ method. “ns” indicates non-significant differences, and gray dashed lines represent the control baseline (fold change = 1). Statistical analysis was performed using one-way ANOVA followed by Tukey’s post hoc test (* *p* < 0.05, ** *p* < 0.01, *** *p* < 0.001 vs. control).

Transcript-level responses to melphalan (15 µM), curcumin (30 µM), and their combination were evaluated in WERI-Rb-1 cells using qRT-PCR. Expression levels of apoptosis-related genes (BAX, BCL2, CASP3, CASP7, CASP9, and SURVIVIN) and STAT3 were normalized to β-actin and GAPDH using the 2^−ΔΔCt^ method. BAX expression increased to 1.8 ± 0.2-fold with curcumin (*p* < 0.05), 2.3 ± 0.3-fold with melphalan (*p* < 0.01), and 3.2 ± 0.5-fold in the combination group (*p* < 0.001), with significantly higher expression compared to single treatments (*p* < 0.01). CASP3 expression increased to 1.9 ± 0.2-fold (curcumin, *p* < 0.05), 2.5 ± 0.3-fold (melphalan, *p* < 0.01), and ~3.5-fold in the combination group (*p* < 0.001). Similarly, CASP7 expression increased to 1.6 ± 0.2-fold (curcumin, *p* < 0.05), 2.6 ± 0.2-fold (melphalan, *p* < 0.01), and 3.7 ± 0.4-fold (combination, *p* < 0.001). CASP9 expression increased to 1.7 ± 0.3-fold (curcumin, *p* < 0.01), 2.4 ± 0.3-fold (melphalan, *p* < 0.01), and 3.3 ± 0.6-fold (combination, *p* < 0.001) (Figure 9A). BCL2 expression was not significantly altered by curcumin (1.3 ± 0.2-fold, *p* > 0.05), but decreased to 0.8 ± 0.1-fold following melphalan treatment (*p* < 0.05), and further declined to 0.6 ± 0.1-fold in the combination group (*p* < 0.01). SURVIVIN expression remained unchanged in the curcumin group (0.9 ± 0.1-fold, *p* > 0.05), but decreased to 0.7 ± 0.1-fold with melphalan (*p* < 0.05) and to 0.4 ± 0.1-fold in the combination group (*p* < 0.001) (Figure 9B). The Bax/Bcl-2 ratio increased progressively from 1.0 ± 0.1 in control cells to 1.4 ± 0.3 (curcumin, *p* < 0.05), 2.9 ± 0.7 (melphalan, *p* < 0.01), and 5.3 ± 2.1 in the combination group (*p* < 0.001), indicating a marked shift toward a pro-apoptotic transcriptional profile (Figure 9C). Curcumin treatment alone did not significantly alter STAT3 expression (1.2 ± 0.2-fold, *p* > 0.05). In contrast, melphalan reduced STAT3 expression to 0.7 ± 0.1-fold (*p* < 0.05), while combination treatment resulted in the most pronounced decrease (0.5 ± 0.1-fold, *p* < 0.001) (Figure 9D). Collectively, these findings indicate coordinated transcriptional modulation of apoptotic and survival pathways; however, these results reflect mRNA-level changes and should be interpreted with caution in the absence of comprehensive protein-level validation.

### 3.5. Enhanced Antitumor Activity of Melphalan–Curcumin Combination in 3D Retinoblastoma Spheroids

**Figure 10:**
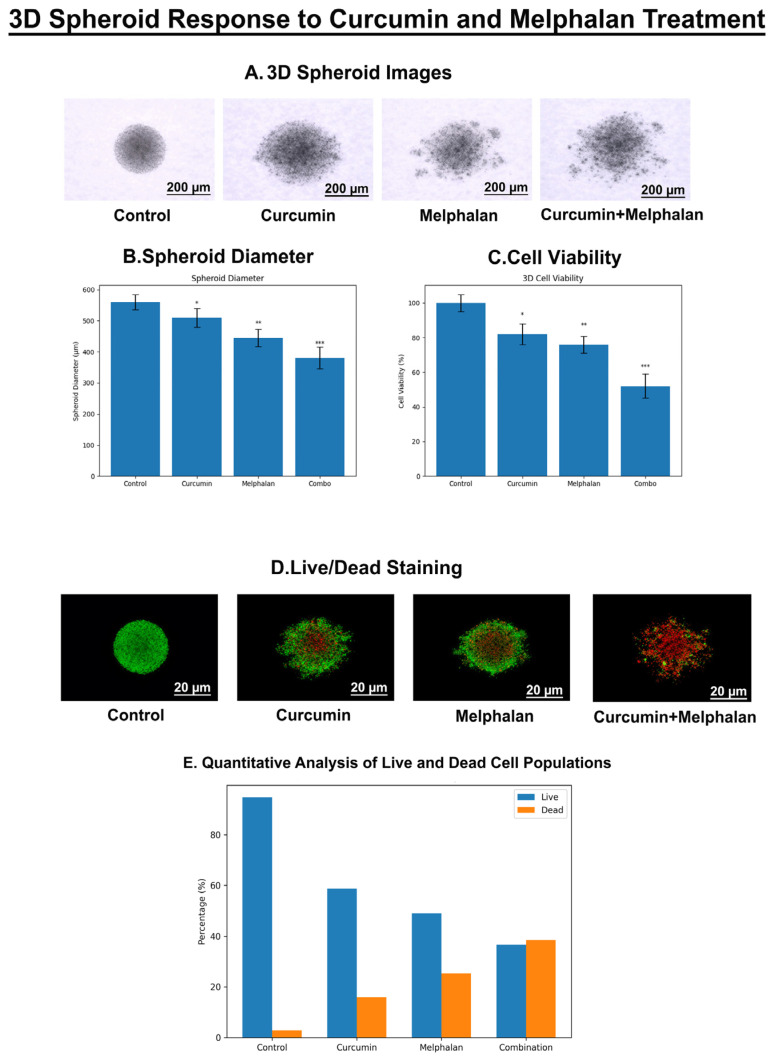
Enhanced antitumor activity of melphalan–curcumin combination in a three-dimensional WERI-Rb-1 retinoblastoma spheroid model. (**A**) Representative bright-field images of 3D tumor spheroids following 72 h treatment with vehicle (control), curcumin, melphalan, or their combination. Control spheroids exhibited compact and well-defined morphology, whereas treated groups showed progressive structural disruption, most prominently in the combination group. Scale bar: 200 µm. (**B**) Quantitative analysis of spheroid diameter. Both single-agent treatments significantly reduced spheroid size compared with control, while the combination treatment resulted in the most pronounced reduction. (**C**) Cell viability of 3D spheroids measured using an ATP-based luminescence assay. Combination treatment produced the strongest reduction in viability compared with both control and single-agent treatments. (**D**) Live/dead fluorescence staining using Calcein-AM (green, viable cells) and Ethidium homodimer-1 (red, dead cells). Control spheroids displayed predominantly green fluorescence, whereas treated spheroids showed increased red signal, with extensive cell death observed in the combination group. Scale bar: 20 µm. Quantitative analysis of live/dead staining is presented alongside representative images. (**E**) Quantitative analysis of live and dead cell populations derived from flow cytometric data, presented as percentage distribution. Due to differences in fluorescence signal intensity and spatial distribution within 3D spheroids, red fluorescence may appear visually dominant despite comparable proportions of live and dead cells. Therefore, imaging results are interpreted qualitatively alongside quantitative analysis. Collectively, these findings suggest that the melphalan–curcumin combination exerts enhanced cytotoxic and growth-inhibitory effects in a three-dimensional tumor model, consistent with preservation of synergistic activity under more physiologically relevant conditions rather than definitive evidence of mechanism. Data are presented as mean ± SD (*n* = 3) for quantitative analyses (**B**,**C**,**E**). Representative images are shown for panels A and D. Statistical analysis was performed using one-way ANOVA followed by Tukey’s post hoc test (* *p* < 0.05, ** *p* < 0.01, *** *p* < 0.001 vs. control).

Under control conditions, WERI-Rb-1 cells formed compact and well-defined three-dimensional spheroids with smooth borders. Curcumin treatment resulted in a modest reduction in spheroid size while largely preserving structural integrity. Melphalan treatment induced more pronounced structural alterations, including partial loss of compactness and early disorganization. In contrast, combination treatment led to marked spheroid shrinkage and substantial architectural disruption, characterized by irregular borders, reduced cellular density, and evident fragmentation, indicating severe impairment of spheroid integrity (Figure 10A).

Quantitative analysis revealed a significant reduction in spheroid diameter following both single-agent treatments compared with control. Notably, the combination group exhibited the most pronounced decrease in spheroid size, significantly lower than both melphalan and curcumin alone (*p* < 0.01–0.001), demonstrating enhanced growth inhibition in the 3D context (Figure 10B).

Assessment of ATP-based viability showed that both melphalan and curcumin significantly reduced spheroid viability relative to control (*p* < 0.05–0.01). Importantly, combination treatment resulted in the strongest reduction in viability (*p* < 0.001 vs. single treatments), indicating a synergistic cytotoxic effect that is preserved in the three-dimensional tumor model (Figure 10C).

Live/dead fluorescence imaging demonstrated predominantly green fluorescence in control spheroids, indicating high cell viability and preserved architecture. Single-agent treatments increased red fluorescence, reflecting moderate induction of cell death. In contrast, combination-treated spheroids exhibited extensive red fluorescence with a marked reduction in green signal, indicating widespread loss of viability and severe structural disruption, consistent with enhanced cytotoxic activity (Figure 10D). Collectively, these findings demonstrate that the enhanced antitumor effects observed in monolayer cultures are maintained and further amplified in a three-dimensional tumor microenvironment, supporting the translational relevance of the melphalan–curcumin combination.

### 3.6. ROS Contributes to, but Does Not Fully Mediate, Melphalan–Curcumin-Induced Cytotoxicity in 3D Spheroids

**Figure 11:**
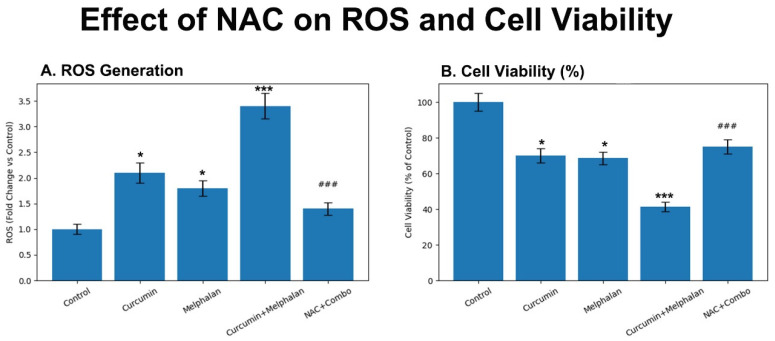
Functional involvement of ROS in melphalan–curcumin-induced cytotoxicity in WERI-Rb-1 retinoblastoma cells. (**A**) Intracellular ROS levels measured by DCFH-DA fluorescence and expressed as fold change relative to control. Both melphalan and curcumin increased ROS levels, while combination treatment induced the highest ROS accumulation. Pretreatment with NAC markedly attenuated ROS levels in the combination group. (**B**) Cell viability assessed following treatment with melphalan, curcumin, and their combination in the presence or absence of NAC. The combination treatment significantly reduced cell viability compared with single treatments, whereas NAC pretreatment partially restored cell viability, suggesting a functional but incomplete rescue effect. Cell viability was calculated as percentage relative to untreated control. Collectively, these findings suggest that ROS generation contributes to, but does not fully account for, the cytotoxic effects of the melphalan–curcumin combination. Data are presented as mean ± SD (*n* = 3). Statistical analysis was performed using one-way ANOVA followed by Tukey’s post hoc test (* *p* < 0.05, *** *p* < 0.001 vs. control; ### *p* < 0.001 vs. combination group).

Treatment of three-dimensional WERI-Rb-1 spheroids with melphalan significantly increased intracellular ROS levels compared with control, while curcumin treatment also induced a moderate elevation in ROS. Notably, combination treatment resulted in the highest ROS accumulation, indicating enhanced oxidative stress under dual-treatment conditions (Figure 11A). Pretreatment with the ROS scavenger N-acetyl-L-cysteine (NAC, 5 mM) significantly reduced ROS levels in the combination group, confirming effective attenuation of oxidative stress.

Consistent with ROS reduction, NAC pretreatment significantly increased cell viability in spheroids exposed to the melphalan–curcumin combination (Figure 11B), indicating a functional rescue effect. However, cell viability in the NAC-pretreated group remained significantly lower than control levels, demonstrating that ROS scavenging only partially reverses combination-induced cytotoxicity. These findings indicate that ROS generation functionally contributes to the cytotoxic effects of the melphalan–curcumin combination, but is not the sole driver, suggesting involvement of additional mechanisms such as mitochondrial apoptotic signaling.

Although NAC-mediated scavenging of ROS significantly reduced oxidative stress and partially restored spheroid viability, the incomplete reversal of cytotoxicity indicates that ROS generation contributes to, but does not fully account for, melphalan + curcumin–induced cell death. This partial rescue effect suggests that ROS acts as a functional mediator rather than the sole driver of cytotoxicity. Instead, the enhanced anticancer activity of the combination appears to arise from the convergence of ROS-dependent stress with additional cell death pathways, particularly mitochondrial apoptotic signaling. This interpretation is supported by the marked increase in caspase activation and the substantial elevation in apoptotic cell fractions observed in flow cytometric analyses. Collectively, these findings support a multifactorial mechanism underlying the enhanced cytotoxic response induced by combined melphalan and curcumin treatment in three-dimensional retinoblastoma tumor spheroids.

### 3.7. Bioinformatic Analysis of Shared Targets of Melphalan and Curcumin

**Figure 12:**
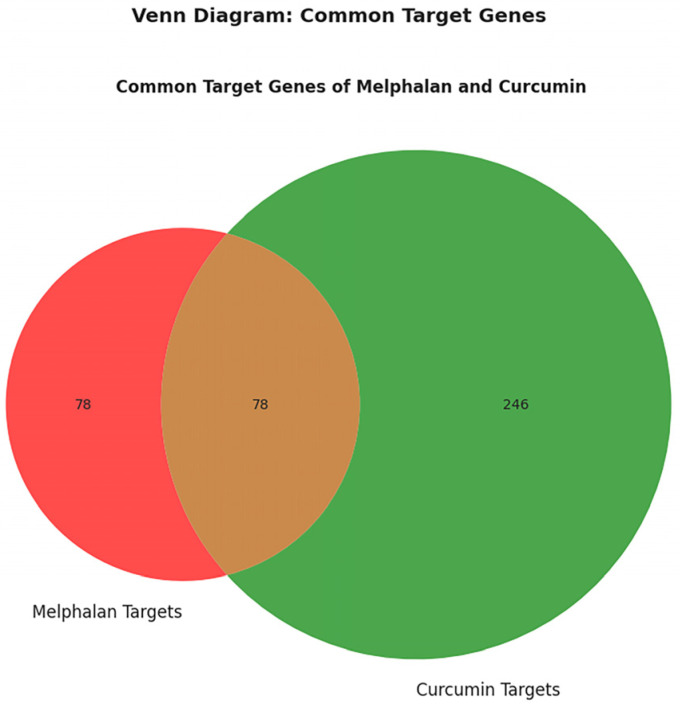
Identification of shared molecular targets of melphalan and curcumin. Venn diagram illustrating the overlap between melphalan-associated genes (*n* = 156; CTD) and curcumin-associated genes (*n* = 324; PubChem and STRING databases). A total of 78 genes were identified as common targets shared by both compounds, representing candidate molecular targets for further network and pathway analyses.

A bioinformatics-based target mining approach was employed to identify molecular targets potentially shared by melphalan and curcumin. Melphalan-associated genes were retrieved from the Comparative Toxicogenomics Database (CTD), yielding 156 candidate targets, while curcumin-related targets (*n* = 324) were obtained from PubChem and STRING databases. Intersection analysis identified 78 common target genes shared between the two compounds (Figure 12). These overlapping targets represent potential molecular nodes through which melphalan and curcumin may exert convergent biological effects. Notably, these shared targets provide a basis for downstream network and pathway analyses, enabling identification of key regulatory hubs and signaling pathways associated with apoptosis, oxidative stress, and STAT3-related signaling.

**Figure 13:**
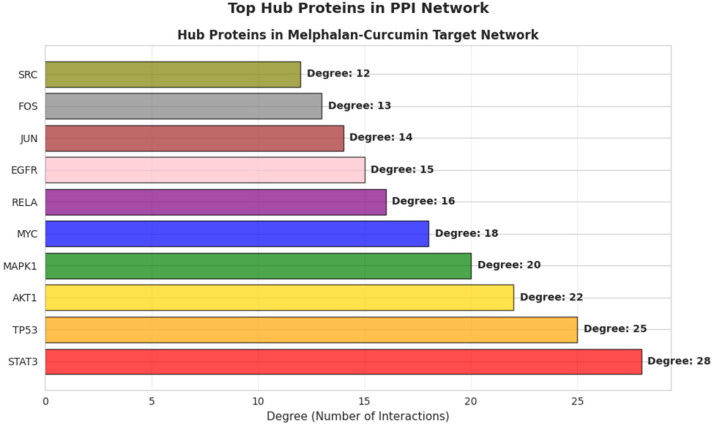
Hub protein analysis in the melphalan–curcumin target network. Bar graph showing the top 10 hub proteins ranked by degree centrality (number of interactions) in the PPI network constructed from shared targets. STAT3 exhibited the highest connectivity, followed by TP53, AKT1, and MAPK1. These highly connected proteins represent potentially associated nodes within the network and may be associated with biological processes relevant to apoptosis, cell survival, and stress-related pathways. The identified hub structure provides a network-level framework suggesting the potential association of STAT3-related and apoptosis-related pathways in the observed effects of melphalan–curcumin co-treatment.

A PPI network was constructed from the 78 shared targets using the STRING database and visualized in Cytoscape. Network topology analysis based on node degree identified ten highly connected hub proteins, including STAT3, TP53, AKT1, MAPK1, MYC, RELA, EGFR, JUN, FOS, and SRC. Among these, STAT3 exhibited the highest degree of connectivity (degree = 28), suggesting a central position within the interaction network (Figure 13). Notably, several identified hub proteins are functionally associated with key cancer-related processes, including apoptosis (TP53, AKT1, MAPK1), transcriptional regulation (MYC, JUN, FOS), and inflammatory signaling (RELA), indicating that the shared target network is enriched in pathways relevant to cell survival and stress responses. These findings highlight STAT3 as a potential network-level regulatory node and provide a rationale for subsequent experimental validation of STAT3-associated signaling and apoptosis-related mechanisms.

**Figure 14:**
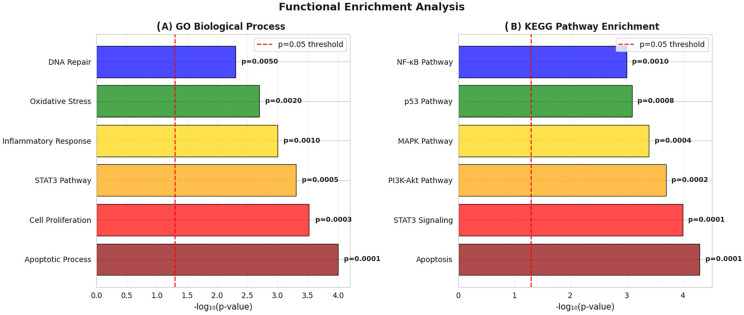
Functional enrichment analysis of shared targets of melphalan and curcumin. (**A**) GO biological process analysis indicating enrichment in apoptosis, cell proliferation, STAT3-related processes, inflammatory response, oxidative stress, and DNA damage repair. (**B**) KEGG pathway enrichment analysis identifying apoptosis, STAT3-related pathways, PI3K–AKT, MAPK, p53, and NF-κB pathways among enriched signaling pathways. All displayed pathways met statistical significance criteria (adjusted *p* < 0.05). Enrichment results are presented as −log10(*p*-value).

GO biological process analysis indicated significant enrichment in pathways related to apoptosis (*p* = 0.0001), cell proliferation (*p* = 0.0003), STAT3 signaling (*p* = 0.0005), inflammatory response (*p* = 0.001), oxidative stress (*p* = 0.002), and DNA damage repair (*p* = 0.005) (Figure 14A). Similarly, KEGG pathway enrichment analysis highlighted multiple cancer-related signaling pathways, including apoptosis (*p* = 0.0001), STAT3 signaling (*p* = 0.0001), PI3K–AKT (*p* = 0.0002), MAPK (*p* = 0.0004), p53 (*p* = 0.0008), and NF-κB (*p* = 0.001) pathways (Figure 14B). Importantly, these enriched pathways are functionally consistent with the experimental findings, particularly the induction of apoptosis, modulation of oxidative stress, and involvement of STAT3-associated signaling observed in melphalan–curcumin-treated retinoblastoma cells. Collectively, these results support a network-level framework in which apoptosis, ROS-related stress responses, and STAT3 signaling pathways may jointly contribute to the observed cytotoxic effects.

**Figure 15:**
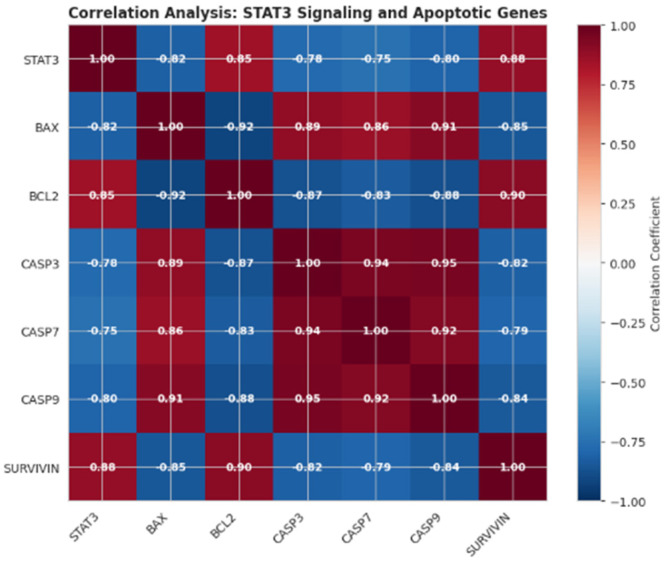
Correlation matrix of STAT3-related transcriptional activity and apoptosis-related genes in retinoblastoma. Heatmap illustrating Pearson correlation coefficients among STAT3 and apoptosis-associated genes (BAX, BCL2, CASP3, CASP7, CASP9, and SURVIVIN). Correlation analysis indicated that STAT3 expression was positively associated with anti-apoptotic genes (BCL2 and SURVIVIN) and negatively associated with pro-apoptotic genes (BAX, CASP3, CASP7, and CASP9), suggesting a potential relationship between STAT3-related transcriptional patterns and apoptotic gene expression. The color scale represents correlation coefficients ranging from −1 (blue, negative correlation) to +1 (red, positive correlation), indicating the direction and strength of gene expression relationships.

Pearson correlation analysis revealed that STAT3 expression showed strong positive correlations with anti-apoptotic genes BCL2 (r = 0.85) and SURVIVIN (r = 0.88). In contrast, STAT3 expression was strongly negatively correlated with pro-apoptotic genes, including BAX (r = −0.82), CASP3 (r = −0.78), CASP7 (r = −0.75), and CASP9 (r = −0.80) (Figure 15). These correlation patterns suggest a coordinated relationship between STAT3 signaling and the balance of pro- and anti-apoptotic gene expression, supporting a potential association between STAT3 activity and cell survival pathways in retinoblastoma.

**Figure 16:**
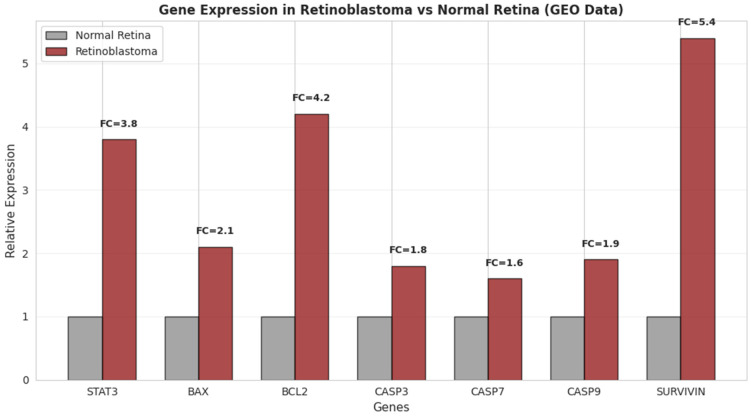
GEO-based transcriptomic validation of gene expression patterns in retinoblastoma. Gene expression profiles derived from publicly available GEO datasets (GSE110811 and GSE97508) comparing retinoblastoma samples with normal retina tissue. Comparative analysis indicated that STAT3 expression was higher (3.8-fold) in retinoblastoma, alongside increased expression of anti-apoptotic genes BCL2 (4.2-fold) and SURVIVIN (5.4-fold). These findings suggest a complex transcriptional profile in retinoblastoma, characterized by concurrent upregulation of survival-associated and apoptosis-related genes rather than direct evidence of pathway activation. Data are presented as relative expression values normalized to normal retina (set as 1.0).

GEO-based transcriptomic validation (GSE110811 and GSE97508) indicated that retinoblastoma samples exhibit elevated expression of STAT3 (3.8-fold), BCL2 (4.2-fold), and SURVIVIN (5.4-fold) compared to normal retina. In addition, pro-apoptotic genes BAX, CASP3, CASP7, and CASP9 also showed moderate increases in expression (2.1-, 1.8-, 1.6-, and 1.9-fold, respectively) (Figure 16). These findings suggest a complex transcriptional landscape in retinoblastoma, characterized by concurrent activation of survival-associated pathways and modulation of apoptosis-related gene expression.

## 4. Discussion

In the current study, we investigated the joint effects of melphalan and curcumin in retinoblastoma using an integrated experimental framework. The combination treatment demonstrated enhanced cytotoxic and pro apoptotic activity compared with single agents, accompanied by increased oxidative stress in conjunction with modulation of apoptosis-associated gene expression. Importantly, these effects were consistently observed in both 2D monolayer cultures systems and three-dimensional spheroid models, supporting the biological relevance of the observed effects. The combined use of two-dimensional and three-dimensional models was designed to balance experimental control with physiological relevance. Overall, the results suggest a multifactorial antitumor response associated with oxidative stress, mitochondrial apoptosis, and STAT3 related transcriptional changes, which should be interpreted within an exploratory preclinical context rather than as definitive mechanistic evidence.

From a methodological perspective, drug concentrations used in the three-dimensional spheroid model were selected based on IC_50_ values derived from two-dimensional dose–response experiments and applied as fixed reference conditions. This approach was intended to ensure consistency across experimental platforms and to facilitate direct comparison between two-dimensional and three-dimensional systems. Importantly, the primary aim of the three-dimensional model was to validate key treatment effects under more physiologically relevant conditions rather than to establish independent dose response relationships. Nevertheless, we acknowledge that optimization of drug concentrations directly within three-dimensional systems would provide additional insight and represents an important direction for future studies.

Mechanistically, our findings indicate that oxidative stress contributes to, but is not solely responsible for, the observed cytotoxic response. The pronounced increase in intracellular ROS following combination treatment, together with the partial rescue of cell viability upon NAC pretreatment, supports a contributory role of ROS while also indicating the involvement of additional cell death pathways. In line with this, co treatment induced a substantial shift toward apoptosis, as confirmed by increased apoptotic fractions, enhanced activation of caspase-7 and caspase-9, and an elevated Bax to Bcl 2 ratio, in line with engagement of the mitochondrial apoptosis pathway. While NAC-based rescue experiments support a functional role of ROS in cytotoxicity, its contribution to other observed effects was not directly evaluated.

Furthermore, network pharmacology and transcriptomic validation analyses converged on STAT3, which emerged as a potentially relevant node associated with both survival and apoptotic gene networks. The observed downregulation of STAT3 expression, together with its inverse relationship with pro apoptotic genes and positive association with anti-apoptotic factors, suggests a possible association between STAT3 related transcriptional changes and the observed pro apoptotic shift, rather than direct evidence of pathway modulation. However, STAT3 activity was not assessed at the protein or phosphorylation level, and therefore conclusions regarding STAT3 signaling remain limited to transcript level observations. Collectively, these findings support a multifactorial context in which ROS modulation, mitochondrial apoptosis, and STAT3 associated transcriptional alterations may act in concert in retinoblastoma cells. Notably, comparison of single-agent and combination treatments revealed both convergent and distinct transcriptional responses. While melphalan primarily induced pro-apoptotic gene expression and reduced STAT3 levels, and curcumin exerted more modest and variable effects, the combined treatment led to a more pronounced and coordinated shift in gene expression patterns. This included enhanced upregulation of pro-apoptotic genes (BAX, CASP3, CASP7, CASP9), stronger suppression of anti-apoptotic regulators (BCL2 and SURVIVIN), and a greater reduction in STAT3 expression compared with either agent alone. These findings suggest that the combined treatment amplifies and integrates the individual effects of melphalan and curcumin, leading to a more robust pro-apoptotic transcriptional profile rather than introducing entirely distinct pathway activation.

The observed synergistic interaction between melphalan and curcumin (CI = 0.78) suggests a complementary mode of action between the two agents. Melphalan, a bifunctional alkylating agent, mediates its cytotoxic effects predominantly through the formation of DNA interstrand crosslinks, leading to replication stress and cell cycle arrest [19]. In contrast, curcumin is a pleiotropic compound known to modulate multiple oncogenic signaling pathways, including those involved in oxidative stress, apoptosis, and inflammation [20]. The convergence of these distinct yet complementary mechanisms may underlie the enhanced cytotoxic response observed in the combination setting. Analogous synergism has been documented for the melphalan–curcumin combination in breast cancer cells [21], and curcumin combined with adriamycin has previously demonstrated cooperative pro-apoptotic activity in retinoblastoma [22]. The favorable selectivity profile observed in HaCaT cells (SI > 3) further supports the preferential cytotoxicity of this combination toward malignant cells [23]. HaCaT cells were used as a non-malignant epithelial reference model for cytotoxicity comparison; however, as they are not derived from retinal tissue, this represents a limitation that should be considered when interpreting selectivity.

An additional consideration for clinical translation is the well-documented low bioavailability of curcumin, which is primarily attributed to its poor solubility, rapid metabolism, and limited systemic distribution [24]. However, recent advances in drug delivery systems have proposed multiple strategies to overcome these limitations. In particular, nanoparticle-based formulations, liposomal carriers, and nanostructured lipid systems have been shown to enhance curcumin stability, permeability, and tissue targeting [25]. In the context of ocular applications, specialized delivery platforms such as nanocarriers and in situ gels have demonstrated improved corneal penetration and retention, supporting the feasibility of targeted intraocular delivery [26]. Therefore, while the present findings are based on in vitro systems, future studies integrating optimized delivery approaches will be essential to improve the translational potential of curcumin-based combination therapies.

Functional assays further demonstrated that co-treatment significantly impaired cellular motility, as evidenced by marked inhibition of wound closure compared with single-agent treatments. Suppression of cell migration is a critical determinant in limiting tumor invasion and metastatic dissemination [27]. Curcumin has previously been shown to inhibit migration in various cancer types [28], including retinoblastoma through modulation of the JAK/STAT signaling pathway and microRNA regulation [29]. The enhanced anti-migratory effect observed in the present study may therefore reflect the combined impact of melphalan-induced cytotoxic stress and curcumin-mediated signaling modulation.

The role of oxidative stress was further substantiated by NAC-based rescue experiments, which demonstrated that ROS scavenging significantly attenuated, but did not fully reverse, combination-induced cytotoxicity. This partial rescue effect indicates that ROS functions as an important mediator, but not the sole driver, of cell death. In line with current literature, NAC is not only a direct scavenger of reactive species but also acts as a precursor for glutathione synthesis and a modulator of intracellular redox balance, with multiple context-dependent mechanisms of action [30]. Rather than exerting a single well-defined effect, NAC has been described as a multifaceted redox regulator, whose biological activity may involve disulfide bond reduction, modulation of thiol-dependent signaling, and indirect enhancement of antioxidant capacity. Therefore, NAC pretreatment in this study was designed to allow intracellular accumulation and stabilization of the redox environment prior to exposure to ROS-inducing agents, rather than to act solely as an immediate scavenger. This broader interpretation supports the conclusion that oxidative stress contributes to, but does not fully account for, the observed cytotoxic response. Curcumin is known to exert pro-oxidant effects at higher concentrations, leading to activation of apoptotic signaling cascades in cancer cells [31], whereas melphalan contributes independently to oxidative stress beyond its alkylation mechanism [32]. Co-exposure to both agents may therefore amplify ROS production in a supra-additive manner, potentially contributing to mitochondrial-associated apoptotic signaling and cell death [33].

Consistent with this, flow cytometric analysis revealed that combination treatment markedly increased total apoptotic cell fractions beyond those observed with individual treatments. This was further supported by increased caspase-7 and caspase-9 activity, as well as enhanced caspase-9 protein expression detected by immunocytochemistry. Activation of caspase-9 is a well-established indicator of intrinsic apoptotic pathway engagement [34,35,36]. In parallel, qRT-PCR analyses demonstrated upregulation of pro-apoptotic genes (BAX, CASP3, CASP7, CASP9) and downregulation of anti-apoptotic genes (BCL2, SURVIVIN), resulting in a marked increase in the Bax/Bcl-2 ratio. This shift in apoptotic balance is widely recognized as a key determinant of mitochondrial commitment to apoptosis [37,38,39].

Beyond its pro-apoptotic activity, the combined treatment modulated inflammatory signaling, as evidenced by reduced levels of IL-6, IL-1β, and TNF-α. Notably, melphalan monotherapy increased cytokine secretion, whereas curcumin alone and more prominently in combination attenuated this response. This anti-inflammatory effect of curcumin is mechanistically linked to its capacity to inhibit NF-κB and STAT3 transcription factors, both of which play key roles in tumor-associated inflammation [40].

Analysis of STAT3 pathway activity demonstrated that the drug combination reduced STAT3 expression at the transcript level. Constitutive activation of STAT3 has been implicated in the progression of retinoblastoma and other malignancies [41]. Through its downstream targets, STAT3 contributes to cell survival, resistance to apoptosis, and inflammatory signaling [42,43].

Bioinformatics analyses further supported the experimental findings by identifying enrichment in apoptosis, oxidative stress, and STAT3-related pathways. The identification of STAT3 as a highly connected hub protein within the PPI network, together with its correlation with apoptosis-related genes and its elevated expression in retinoblastoma datasets, underscores its potential relevance as a network-level associated node rather than a functionally validated regulator. However, these in silico findings should be interpreted as hypothesis-generating and require further experimental validation. In the present study, bioinformatics analysis was intentionally positioned as a complementary approach to reinforce experimentally derived observations rather than serving as a predictive framework or hypothesis-generating starting point.

**Figure 17:**
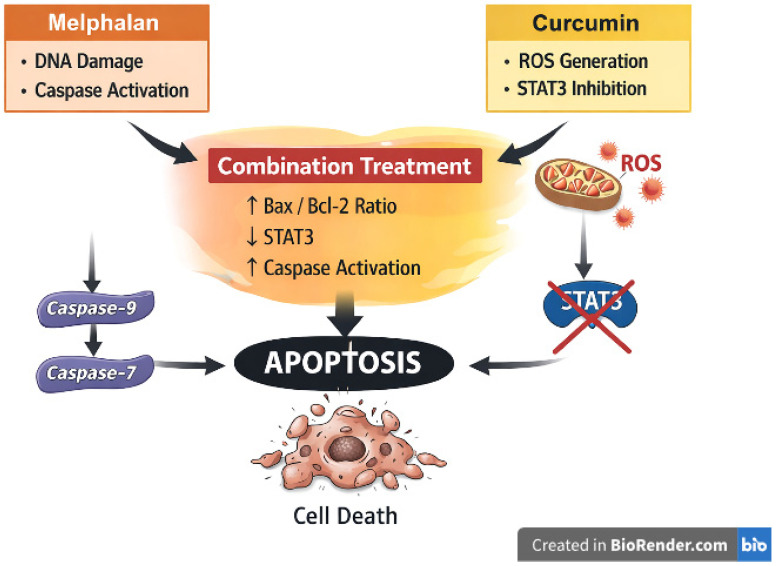
Schematic overview of the proposed mechanisms underlying the combined effects of melphalan and curcumin in retinoblastoma cells. Melphalan and curcumin act through partially distinct but converging biological processes, including DNA damage-associated stress, oxidative stress, and modulation of survival-related signaling. Their combined application is associated with enhanced apoptotic signaling, characterized by increased caspase activation, an elevated Bax/Bcl-2 ratio, and reduced STAT3 expression. These coordinated effects suggest a multifactorial shift toward apoptosis rather than reliance on a single dominant pathway.

Collectively, the integrated experimental and in silico findings support a multifactorial model underlying the enhanced cytotoxic effects of melphalan–curcumin co-treatment in retinoblastoma cells. In this framework, melphalan primarily contributes through DNA damage-associated stress and activation of the intrinsic apoptotic pathway, while curcumin is associated with increased intracellular ROS generation and modulation of STAT3-related signaling. When combined, these effects appear to converge, resulting in amplified oxidative stress, increased Bax/Bcl-2 ratio, enhanced caspase activation, and suppression of survival-associated transcriptional programs. Importantly, the observed effects do not indicate a single dominant pathway but rather suggest a coordinated interaction between ROS-mediated stress, mitochondrial apoptotic signaling, and STAT3-associated transcriptional regulation. A schematic representation of this proposed mechanism is provided in Figure 17.

Several limitations of this study should be acknowledged. In addition, the use of a single retinoblastoma cell line (WERI-Rb-1) represents a limitation, as it may not fully capture the biological heterogeneity of retinoblastoma tumors. First, the findings are based on in vitro models, including both monolayer and three-dimensional spheroid systems, which may not fully recapitulate the complexity of in vivo tumor biology. Second, although multiple complementary assays were employed to assess cytotoxicity, oxidative stress, and apoptosis, comprehensive protein-level validation, such as cleaved caspase, PARP, phosphorylated STAT3, and DNA damage markers, was not performed. In particular, the distinction between cleaved and total caspase-9 was not assessed, and therefore conclusions regarding caspase-9 activation remain limited. Moreover, STAT3 activity was not evaluated at the protein level, and phosphorylated STAT3 (p-STAT3) relative to total STAT3 was not assessed, which limits conclusions regarding STAT3 pathway activation. Third, direct functional interrogation of key pathways, including genetic or pharmacological modulation of STAT3 and apoptosis signaling, was not included. Fourth, proliferation was not directly assessed using specific markers such as Ki67, and the antiproliferative effects observed in this study are therefore based on indirect functional readouts, particularly in the three-dimensional spheroid model. Fifth, NAC treatment was not systematically applied across all experimental assays, and therefore the role of ROS in processes such as migration, apoptosis, cytokine regulation, gene expression, and three-dimensional tumor behavior remains only partially defined. Sixth, migration analysis was limited to scratch wound assays, and additional methods such as transwell invasion assays were not performed. Seventh, the use of HaCaT cells as a non-malignant control, while providing a reproducible epithelial reference, does not fully represent normal retinal tissue. In addition, the use of cell lines with inherently different growth characteristics introduces a potential source of variability. Finally, drug concentrations used in three-dimensional experiments were extrapolated from two-dimensional conditions and not independently optimized under spheroid-specific conditions. Therefore, the findings related to STAT3 should be interpreted as transcriptional associations rather than direct evidence of functional pathway activation. These limitations indicate that the present findings should be interpreted as exploratory and hypothesis-generating, and further in vivo and mechanistic studies are required to confirm the proposed associations.

## 5. Conclusions

In conclusion, the present study demonstrates that melphalan and curcumin co-treatment exerts synergistic antiproliferative and pro-apoptotic effects in retinoblastoma cells through a multifactorial mechanism potentially associated with ROS-related effects, mitochondrial apoptotic signaling, and STAT3-associated transcriptional changes.

Mechanistic investigations, including NAC-based rescue experiments, indicate that ROS generation contributes to, but does not fully account for, the observed cytotoxic effects, highlighting the possible involvement of additional pathways such as intrinsic apoptosis and survival-related processes. The integration of experimental and bioinformatics analyses further supports a coordinated association between oxidative stress, apoptotic regulation, and STAT3-related transcriptional activity, rather than definitive pathway modulation.

Moreover, the combination exhibited preferential cytotoxicity toward tumor cells compared with non-malignant cells, suggesting a favorable therapeutic window.

Taken together, these findings position melphalan–curcumin co-treatment as a promising preclinical strategy for retinoblastoma. However, further in vivo validation, mechanistic clarification, and pharmacokinetic optimization are required to determine its translational potential.

## Figures and Tables

**Table 1:** Primer sequences used for quantitative real-time PCR analysis.

Gene	Forward Primer (5′→3′)	Reverse Primer (5′→3′)
BAX	TCAGGATGCGTCCACCAAGAAG	TGTGTCCACGGCGGCAATCATC
BCL2	ATCGCCCTGTGGATGACTGAGT	GCCAGGAGAAATCAAACAGAGGC
CASP3	AGAGGGGATCGTTGTAGAAGCTG	CACAAGCGACTGGATGAACCA
CASP7	CCTCATCATCAACAACCTGG	AAGTCCCTTTCGCAGAAACAG
CASP9	CCGTCCATGCGGAAGATC	ATGGCCAGCGGGAAGAC
SURVIVIN	GGAAACCAGGAAGCCTAGCATC	GGATGATTCAGTGCCATTTTGCC
STAT3	CTTTGAGACCGAGGTGTATCACC	GGTCAGCATGTTGTACCACAGG
ACTB (β-actin)	CATTGCTGACAGGATGCAGAAGG	TGCTGGAAGGTGGACAGTGAGG
GAPDH	GGAGCGAGATCCCTCCAAAAT	GGCTGTTGTCATACTTCTCATGG

## Data Availability

All data that support the findings from this study are available from the corresponding author upon request.

## Abbreviations

ACTB	Beta-actin
ANOVA	Analysis of Variance
ATP	Adenosine Triphosphate
BAX	BCL2 Associated X Protein
BCL2	B-cell Lymphoma 2
BCA	Bicinchoninic Acid
CASP3	Caspase-3
CASP7	Caspase-7
CASP9	Caspase-9
CI	Combination Index
CTD	Comparative Toxicogenomics Database
DAVID	Database for Annotation, Visualization and Integrated Discovery
DCFH-DA	2′,7′-Dichlorodihydrofluorescein Diacetate
DMSO	Dimethyl Sulfoxide
ELISA	Enzyme-Linked Immunosorbent Assay
FBS	Fetal Bovine Serum
FITC	Fluorescein Isothiocyanate
GAPDH	Glyceraldehyde-3-Phosphate Dehydrogenase
GEO	Gene Expression Omnibus
GO	Gene Ontology
HRP	Horseradish Peroxidase
IC_50_	Half Maximal Inhibitory Concentration
IOD	Integrated Optical Density
KEGG	Kyoto Encyclopedia of Genes and Genomes
MTT	3-(4,5-Dimethylthiazol-2-yl)-2,5-Diphenyltetrazolium Bromide
NAC	N-Acetyl-L-Cysteine
PBS	Phosphate-Buffered Saline
PI	Propidium Iodide
PPI	Protein–Protein Interaction
qRT-PCR	Quantitative Real-Time Polymerase Chain Reaction
ROS	Reactive Oxygen Species
SD	Standard Deviation
SI	Selectivity Index
STAT3	Signal Transducer and Activator of Transcription 3
STRING	Search Tool for the Retrieval of Interacting Genes/Proteins
WERI-Rb-1	Human Retinoblastoma Cell Line

## References

[1] Nag A., Khetan V. (2024). Retinoblastoma—A comprehensive review, update and recent advances. Indian J. Ophthalmol..

[2] Gersey Z.C., Rodriguez G.A., Barbarite E., Sanchez A., Walters W.M., Ohaeto K.C., Komotar R.J., Graham R.M. (2017). Curcumin decreases malignant characteristics of glioblastoma stem cells via induction of reactive oxygen species. BMC Cancer.

[3] Redondo-Villanueva M.J.H., Mercado G.J.V. (2022). Intravitreal melphalan for persistent retinoblastoma vitreous seeds. GMS Ophthalmol. Cases.

[4] Bogan C.M., Pierce J.M., Doss S.D., Tao Y.K., Chen S.C., Boyd K.L., Liao A., Hsieh T., Abramson D.H., Francis J.H. (2021). Intravitreal melphalan hydrochloride vs. propylene glycol-free melphalan for retinoblastoma vitreous seeds. Exp. Eye Res..

[5] He X., Han M., Zhou M., Chai P., Guo L., Fan J., Wen X., Fan X. (2025). Effect of intra-arterial chemotherapy drug regimens on globe salvage outcomes of retinoblastoma patients. Br. J. Ophthalmol..

[6] Alshahrani N.O., Aldhawi A., Feng Z.X., Chau K., Mallipatna A., Muthusami P., Parra-Farinas C., Zaarour C., Shaikh F., Gallie B.L. (2025). Single- versus triple-agent intra-arterial chemotherapy for retinoblastoma. Am. J. Ophthalmol..

[7] Sen M., Munier F.L., Honavar S.G. (2026). Intraocular seeds in retinoblastoma: A review of classification, management, and outcomes. Surv. Ophthalmol..

[8] Francis J.H., Brodie S.E., Marr B., Zabor E.C., Mondesire-Crump I., Abramson D.H. (2017). Efficacy and toxicity of intravitreous chemotherapy for retinoblastoma: Four-year experience. Ophthalmology.

[9] Weinberger Y., Kapoor S., Zabor E.C., Singh A.D. (2025). Intra-arterial chemotherapy for retinoblastoma: A performance analysis. Br. J. Ophthalmol..

[10] Wong E.S., Choy R.W., Zhang Y., Chu W.K., Chen L.J., Pang C.P., Yam J.C. (2022). Global retinoblastoma survival and globe preservation: A systematic review and meta-analysis. Lancet Glob. Health.

[11] Younes M., Mardirossian R., Rizk L., Fazlian T., Khairallah J.P., Sleiman C., Naim H.Y., Rizk S. (2022). The synergistic effects of curcumin and chemotherapeutic drugs. Plants.

[12] Joshi P., Joshi S., Semwal D., Bisht A., Paliwal S., Dwivedi J., Sharma S. (2021). Curcumin: An insight into molecular pathways involved in anticancer activity. Mini Rev. Med. Chem..

[13] Özdemir İ., Zaman F., Doğan Baş D., Sari U., Öztürk Ş., Tuncer M.C. (2025). Inhibitory effect of curcumin on cervical cancer via RAS/RAF pathway. Histol. Histopathol..

[14] Çavuş Y., Alkan Akalın S., Afşin Y., Toprak V., Özdemir İ., Tuncer M.C., Öztürk Ş. (2026). Synergistic antitumor effects of etoposide and curcumin in ovarian cancer cells. Biomedicines.

[15] Doğan S., Tuncer M.C., Özdemir İ. (2025). Synergistic effect of curcumin and adriamycin on apoptosis in retinal tumor cells. Folia Morphol..

[16] He Y., Liao Y., Fang S., Zhu L., Zhao Z., Chen T., Zhang Z. (2025). Mechanism of Curcumin in Inhibiting Proliferation of Head and Neck Squamous Cell Carcinoma: A Network Pharmacology and Cellular Experimental Study. Biomed. Res. Int..

[17] Akter K., Gul K., Mumtaz S. (2025). Revisiting curcumin in cancer therapy. Curr. Issues Mol. Biol..

[18] Li Y., Sun W., Han N., Zou Y., Yin D. (2018). Curcumin inhibits retinoblastoma via miR-99a/JAK-STAT pathway. BMC Cancer.

[19] Amin S., Rizvi F., Zia N., Ali A., Hamid A., Kumari B. (2020). Outcomes of group D retinoblastoma with resistant vitreous seeds. Cureus.

[20] Patel S.S., Acharya A., Ray R.S., Agrawal R., Raghuwanshi R., Jain P. (2020). Molecular mechanisms of curcumin in disease. Crit. Rev. Food Sci. Nutr..

[21] Shehzad A., Lee Y.S. (2013). Molecular mechanisms of curcumin action: Signal transduction. BioFactors.

[22] Ashrafizadeh M., Zarrabi A., Hashemi F., Zabolian A., Saleki H., Bagherian M., Azami N., Bejandi A.K., Hushmandi K., Ang H.L. (2020). Polychemotherapy with curcumin and doxorubicin. Pharmaceutics.

[23] Zhu X., Wang K., Yao Y., Zhang K., Zhou F., Zhu L. (2018). p53 activation in retinoblastoma apoptosis. J. Biochem. Mol. Toxicol..

[24] Ribeiro A., Oliveira D., Cabral-Marques H. (2025). Curcumin in Ophthalmology: Mechanisms, Challenges, and Emerging Opportunities. Molecules.

[25] Yakubu J., Pandey A.V. (2024). Innovative Delivery Systems for Curcumin: Exploring Nanosized and Conventional Formulations. Pharmaceutics.

[26] Lakhani P., Patil A., Taskar P., Ashour E., Majumdar S. (2018). Curcumin-loaded Nanostructured Lipid Carriers for Ocular Drug Delivery: Design Optimization and Characterization. J. Drug Deliv. Sci. Technol..

[27] M R., Jose S.P., Im K., Sukumaran S., Saji S., Sreevallabhan S. (2021). Curcumin-galactomannoside complex inhibits the proliferation of human cervical cancer cells: Possible role in cell cycle arrest and apoptosis. Asian Pac. J. Cancer Prev..

[28] Tomar A.S., Finger P.T., Gallie B., Kivelä T.T., Mallipatna A., Zhang C., Zhao J., Wilson M.W., Brenna R.C., Burges M. (2021). Global retinoblastoma outcomes. Ophthalmology.

[29] Cao J.F., Zhang X., Xia Q., Hang K., Men J., Tian J., Liao D., Xia Z., Li K. (2025). Curcumin targets in pancreatic cancer. Bioorg. Chem..

[30] Pedre B., Barayeu U., Ezeriņa D., Dick T.P. (2021). The mechanism of action of N-acetylcysteine (NAC): The emerging role of H_2_S and sulfane sulfur species. Pharmacol. Ther..

[31] Wang M., Jiang S., Zhou L., Yu F., Ding H., Li P., Zhou M., Wang K. (2019). Mechanisms of curcumin in cancer prevention. Int. J. Biol. Sci..

[32] Jia F., Peng Y., Li X., Yang S., Xie Y., Han Y., Huang M., Liu T., Zou W., Chen L. (2025). Matrix metallopeptidase 2-responsive curcumin-loaded nanoparticles-induced STAT3 inhibition suppresses glioblastoma multiforme growth via enhancing Nrf2 activity. Int. J. Biol. Macromol..

[33] Das A., Saiteja K., Shah P.K., Subramaniam P., Venkatapathy N. (2025). Intra-arterial chemotherapy real-world data. Int. Ophthalmol. Clin..

[34] Guo T., Wu C., Zhou L., Zhang J., Wang W., Shen Y., Zhang L., Niu M., Zhang X., Yu R. (2023). Mito-LND in glioblastoma. J. Transl. Med..

[35] Sreenivasan S., Krishnakumar S. (2015). Curcumin synergy in retinoblastoma. Curr. Eye Res..

[36] Sreenivasan S., Thirumalai K., Danda R., Krishnakumar S. (2012). Curcumin and miRNA in retinoblastoma. Curr. Eye Res..

[37] Wu C., Zheng W., Zhang J., He X. (2022). Mechanism of curcumin in retinoblastoma. Evid. Based Complement. Alternat. Med..

[38] Chen Q., Zhang B., Dong Y., Mo X., Zhang L., Xia J., Zhang J., Zhang S. (2019). Intra-arterial chemotherapy in infants. BMC Cancer.

[39] Wang K., Tian T., Chen Z., He X., Xiang Y., Zuo G., Cheng S. (2025). Curcumin in ophthalmic diseases. J. Transl. Med..

[40] Peng Y., Ao M., Dong B., Jiang Y., Yu L., Chen Z., Hu C., Xu R. (2021). Anti-inflammatory effects of curcumin in inflammatory diseases: Status, limitations and countermeasures. Drug Des. Devel. Ther..

[41] Abramson D.H., Francis J.H., Knopman J., Dunkel I.J., Gobin Y.P. (2025). Ophthalmic artery chemosurgery in infants. Ophthalmol. Retina.

[42] Pu J., Wang B., Zhang D., Wang K., Yang Z., Zhu P., Song Q. (2024). UBE2T mediates SORBS3 ubiquitination to enhance IL-6/STAT3 signaling and promote lung adenocarcinoma progression. J. Biochem. Mol. Toxicol..

[43] Xu N., Cui Y., Shi H., Guo G., Sun F., Jian T., Rao H. (2022). UBE2T/STAT3 signaling promotes the proliferation and tumorigenesis in retinoblastoma. Invest. Ophthalmol. Vis. Sci..

